# Monocyte-released HERV-K dUTPase engages TLR4 and MCAM causing endothelial mesenchymal transition

**DOI:** 10.1172/jci.insight.146416

**Published:** 2021-08-09

**Authors:** Shoichiro Otsuki, Toshie Saito, Shalina Taylor, Dan Li, Jan-Renier Moonen, David P. Marciano, Rebecca L. Harper, Aiqin Cao, Lingli Wang, Maria E. Ariza, Marlene Rabinovitch

**Affiliations:** 1 Department of Pediatrics, Division of Cardiology, Vera Moulton Wall Center for Pulmonary Vascular Disease, and Cardiovascular Institute, and; 2 Department of Genetics and Cardiovascular Institute, Stanford University School of Medicine, Stanford, California, USA.; 3 Department of Cancer Biology and Genetics, and Institute for Behavioral Medicine Research, The Ohio State University Wexner Medical Center, Columbus, Ohio, USA.

**Keywords:** Inflammation, Vascular Biology, Endothelial cells, Monocytes

## Abstract

We previously reported heightened expression of the human endogenous retroviral protein HERV-K deoxyuridine triphosphate nucleotidohydrolase (dUTPase) in circulating monocytes and pulmonary arterial (PA) adventitial macrophages of patients with PA hypertension (PAH). Furthermore, recombinant HERV-K dUTPase increased IL-6 in PA endothelial cells (PAECs) and caused pulmonary hypertension in rats. Here we show that monocytes overexpressing HERV-K dUTPase, as opposed to GFP, can release HERV-K dUTPase in extracellular vesicles (EVs) that cause pulmonary hypertension in mice in association with endothelial mesenchymal transition (EndMT) related to induction of SNAIL/SLUG and proinflammatory molecules IL-6 as well as VCAM1. In PAECs, HERV-K dUTPase requires TLR4-myeloid differentiation primary response–88 to increase *IL-6* and *SNAIL/SLUG*, and HERV-K dUTPase interaction with melanoma cell adhesion molecule (MCAM) is necessary to upregulate *VCAM1*. TLR4 engagement induces p-p38 activation of NF-**κ**B in addition to p-pSMAD3 required for *SNAIL* and pSTAT1 for *IL-6*. HERV-K dUTPase interaction with MCAM also induces p-p38 activation of NF-**κ**B in addition to pERK1/2-activating transcription factor-2 (ATF2) to increase *VCAM1*. Thus in PAH, monocytes or macrophages can release HERV-K dUTPase in EVs, and HERV-K dUTPase can engage dual receptors and signaling pathways to subvert PAEC transcriptional machinery to induce EndMT and associated proinflammatory molecules.

## Graphical abstract

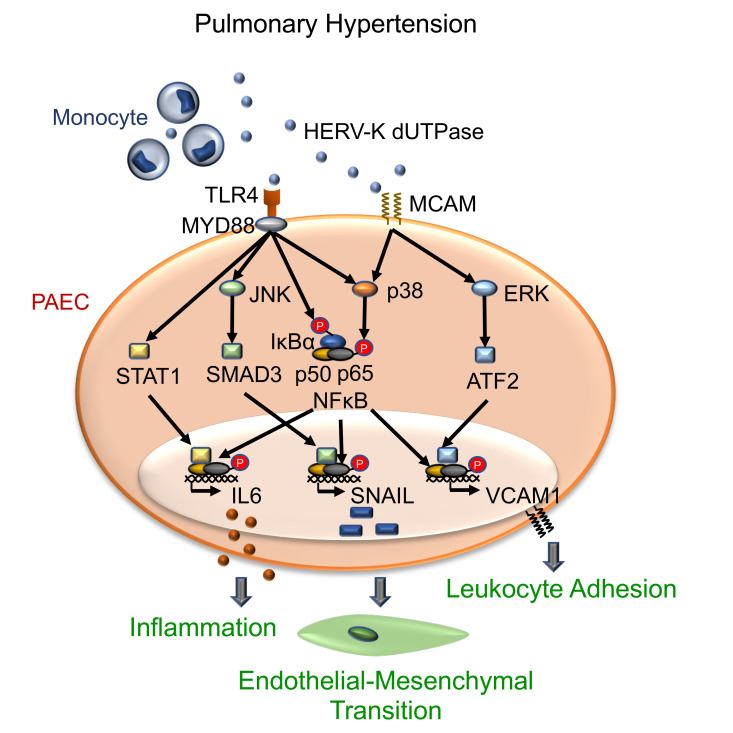

## Introduction

Pulmonary arterial hypertension (PAH) is a progressive disease characterized by obstructive intimal and plexiform lesions in small pulmonary arteries (PAs) that cause increased resistance to flow. While current vasodilator treatments can improve quality of life and increase survival, they do not reverse the pathologic vascular changes that culminate in right ventricular failure and premature death (reviewed in ref. 1). The proliferative expansion of dedifferentiated smooth muscle cells (SMCs) causing the obstructive neointima is, at least in part, the consequence of pulmonary arterial endothelial cell (PAEC) dysfunction. PAH PAECs undergo endothelial mesenchymal transition (EndMT) and, as a result, gain proinflammatory and lose barrier properties (2–4) and may fail to produce inhibitors of SMC proliferation such as apelin (5). Many factors can contribute to EndMT, including loss of function of bone morphogenetic protein receptor-2 (BMPR2), a gene that is mutated in most patients with familial PAH and in 20% of those without a family history of PAH (6). Viruses such as CoxsackieB3 have also been implicated in EndMT in the heart (7).

Human endogenous retrovirus (HERV) sequences are remnants of ancient viruses that comprise 8% of the human genome (8). The majority of HERVs are inactivated by mutations precluding production of a replicating virus. While HERVs are highly expressed in embryonic stem cells, transcription and translation are repressed in differentiated cells (9). However, under certain circumstances, these control mechanisms can be subverted, leading to the production of double-stranded (ds) *HERV* RNA and HERV proteins linked to the pathology of cancer, autoimmune disease (10–13), and most recently, PAH (14). The production of dsRNA induces a chronic interferon response, and HERV proteins, such as the HERV-K envelope and deoxyuridine triphosphate nucleotidohydrolase (dUTPase), can trigger innate as well as adaptive immune responses (15, 16). The relationship between the induction of innate and adaptive immunity and the pathogenesis of PAH has been reviewed (17). Recent studies from our group reported an increase in HERV-K proteins in circulating monocytes and in macrophages located in the adventitia of PAs in patients with PAH. We showed that recombinant HERV-K dUTPase can induce IL-6 production in PAECs as well as the activation of B cells and pulmonary hypertension in rats (14).

Taken together, we postulated that release of HERV-K dUTPase from PAH monocytes and macrophages could induce EndMT associated with a proinflammatory phenotype in PAECs. We first show that HERV-K dUTPase can be packaged in extracellular vesicles (EVs) that replicate the effects of recombinant HERV-K dUTPase in inducing pulmonary hypertension in mice, now shown associated with EndMT and proinflammatory markers. We then demonstrate how HERV-K dUTPase interacts with dual endothelial cell (EC) receptors, TLR4 and melanoma cell adhesion molecule (MCAM), to induce complementary signaling pathways that activate distinct transcription factors required to regulate genes important in EndMT (*SNAIL/SLUG*) and associated inflammation (*IL-6* and *VCAM1*).

## Results

### Recombinant HERV-K dUTPase upregulates SNAIL and induces EndMT in PAECs.

**Figure 1:**
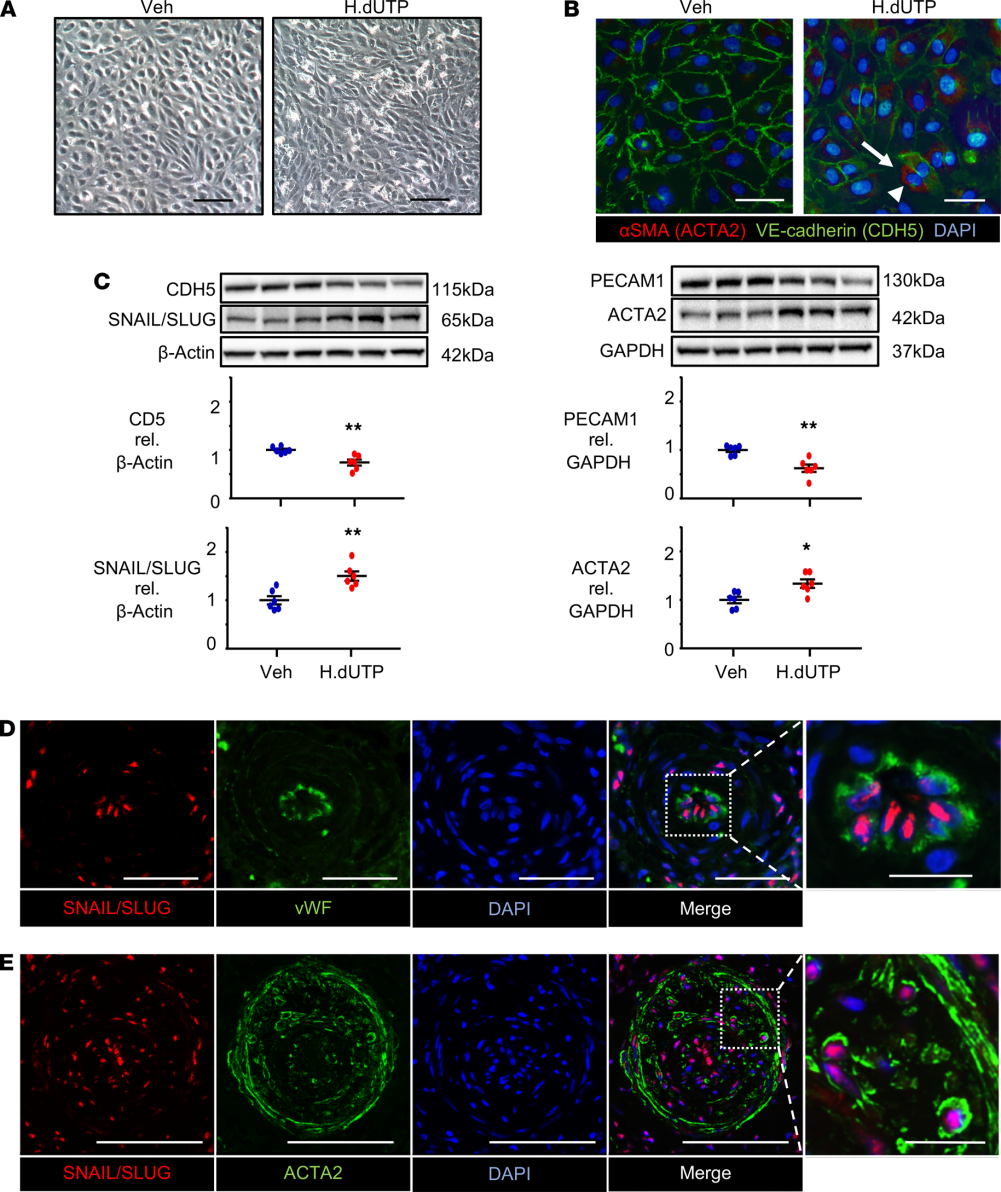
Recombinant HERV-K dUTPase upregulates SNAIL and induces features of EndMT in PAECs. PAECs used at passage 3–8 were treated with 10 μg/mL HERV-K dUTPase (H.dUTP) or with PBS vehicle (Veh) daily for 3 days, then every 3 days up to 10 days. (**A**) Representative phase-contrast light microscopic images, showing the typical cobblestone morphology of control PAECs treated with vehicle versus an elongated spindle shape morphology of HERV-K dUTPase treated PAECs. Scale bar: 100 μm. (**B**) Immunofluorescence microscopy images showing elongated PAECs expressing αSMA (ACTA2) (red) (arrowhead) and greatly diminished VE-cadherin (CDH5) (green) (arrow) in the HERV-K dUTPase-treated PAECs. Scale bar: 50 μm. (**C**) Representative immunoblot and densitometric analysis of protein expression normalized to β-Actin or GAPDH, assessed at 10 days. Individual data points are shown, with *n* = 6, mean ± SEM. *P < 0.05; **P < 0.01 versus Veh, by unpaired Student’s *t* test. (**D**) Representative confocal images of a small PA from 1 of 2 patients with PAH stained for the EndMT transcription marker SNAIL/SLUG (red), the endothelial marker vWF (green), and DAPI (blue), showing SNAIL/SLUG localized to vWF positive PAECs in occlusive lesions (Control in Supplemental Figure 1C). Scale bar: 20 μm (10 μm in magnified panel, far right). (**E**) Representative confocal image of a large PA stained for SNAIL/SLUG (red), ACTA2 as a SMC marker (green), and DAPI (blue), showing SNAIL/SLUG localized to ACTA2-positive cells in an occlusive lesion from 1 of 2 patients with PAH. Scale bar: 50 μm (10 μm in magnified panel, far right).****

We investigated whether HERV-K dUTPase can induce EndMT, a feature increasingly linked to the structural remodeling of PAH (3, 4, 18). Commercially available human PAECs were treated with 10 μg/mL of recombinant HERV-K dUTPase or PBS vehicle once daily for 3 days and every 3 days thereafter for up to 20 days. At day 10 after HERV-K dUTPase, but not vehicle, the morphology of PAECs changed from the typical cobblestone appearance to an elongated spindle shape (Figure 1A). Some of these elongated cells stained positive for the SMC marker ACTA2, consistent with a switch to a mesenchymal phenotype in response to HERV-K dUTPase. There was also a marked reduction in immunostaining for the vascular endothelial-cadherin (VE-cadherin; CDH5), with this EC marker appearing fragmented or even undetectable in some of the cells treated with HERV-K dUTPase (Figure 1B). It was clear that some cells appeared to show more striking changes than others, suggesting that a subset of PAECs may be more susceptible than others to EndMT. We then determined the time course of EndMT in response to HERV-K dUTPase (Supplemental Figure 1A; supplemental material available online with this article; https://doi.org/10.1172/jci.insight.146416DS1). An increase in mRNA levels of the transcription factors associated with EndMT was observed on day 1 for *SNAIL* (*SNAI1*) and day 3 for *SLUG* (*SNAI2*). Reduced levels of EC transcripts were observed for *PECAM1* on day 3 and *CDH5* on day 20, whereas an elevation in SMC transcripts was seen on day 10 for *ACTA2* and on day 20 for *SM22* (*TAGLN*). We did not observe a significant change in mRNA levels of other transcription factors associated with EndMT, such as *TWIST1* (3) and *ZEB1* (19). Based on this time course, we chose day 10 to assess protein levels of the transcripts associated with EndMT and confirmed a HERV-K dUTPase-mediated increase in SNAIL/SLUG (using an antibody that recognizes both transcription factors) and ACTA2 and a reduction in PECAM1 and CDH5 (Figure 1C). We also showed that HERV-K induces initial apoptosis in PAECs followed by resistance to apoptosis, a feature of EndMT (Supplemental Figure 1B). This was reported in experimental rat models of PAH, with reduced BMPR2 and inflammation (18) and with senescence induced by an aortocaval shunt and monocrotaline, and validated in human tissue from patients with PAH by elevated survivin expression (20).

Previous studies have shown an increase in SNAIL mRNA and immunofluorescence in PAH versus control PAECs (21), but it had not, to our knowledge, been documented in PAH versus control lung tissues. We therefore confirmed an increase in SNAIL/SLUG in PAECs in lung tissue sections from patients with idiopathic PAH (IPAH) versus controls. SNAIL/SLUG was observed in the nuclei of vWF-positive PAECs (Figure 1D) as well as in some ACTA2-positive cells in the neointima of occlusive lesions from patients with IPAH (Figure 1E) but it was not detected in PAs from unused donor control subjects (Supplemental Figure 1C). Demographics and other clinical data of the IPAH and control subjects from whom the tissue was used in these analyses can be found in Supplemental Tables 1 and 2.

### EndMT is induced in PAECs cocultured with THP-1 monocytes overexpressing HERV-K dUTPase.

**Figure 2:**
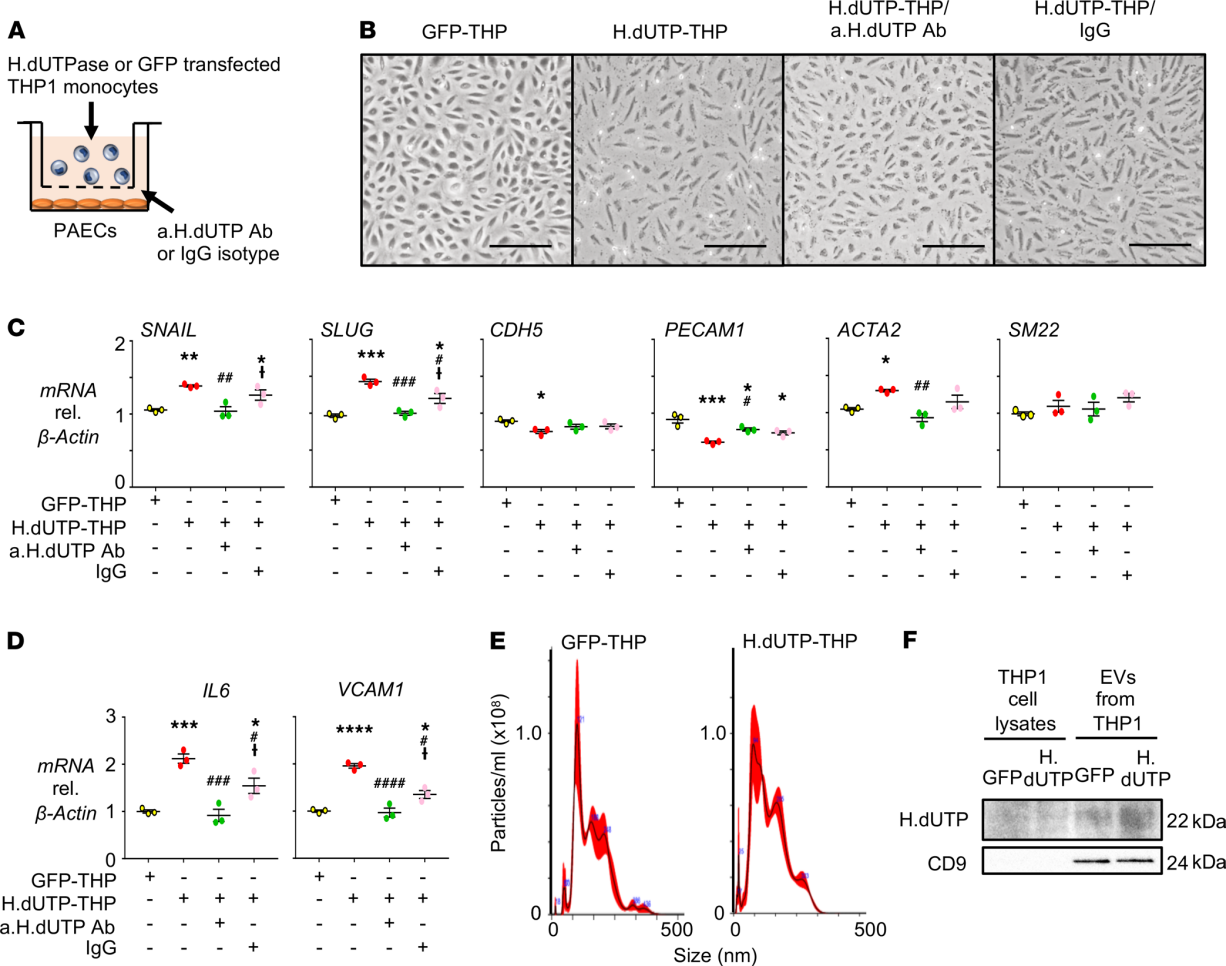
Coculture of PAECs with THP-1 monocytes expressing HERV-K dUTPase reveals features of EndMT. (**A**) Schematic of the coculture experiment. PAECs were cocultured with THP-1 monocytes transfected with HERV-K dUTPase or GFP (H.dUTP-THP or GFP-THP, respectively) in a transwell apparatus with a 1 μm pore membrane. To assess the specific contribution of secreted HERV-K dUTPase, PAECs were pretreated with 10 μg/mL of anti–HERV-K dUTPase neutralizing antibody (a.H.dUTP Ab) or IgG isotype (IgG) for 1 hour before the start of the coculture. Cells were maintained in coculture for 3 days. (**B**) Representative phase-contrast light microscopic images following 3 days in culture show a typical cobblestone morphology of control PAECs cocultured with GFP-THP versus an elongated spindle shape morphology of PAECs cocultured with H.dUTP-THP. Pretreatment of PAEC with a.H.dUTP Ab suppressed the PAEC EndMT morphological changes. Scale bar: 100 μm. (**C**) Gene expression levels of EndMT, EC, and SMC markers in PAECs assessed by qPCR. (**D**) Gene expression levels of *IL-6* and *VCAM1* in PAECs, assessed by qPCR. (**E**) EVs released from GFP-THP or H.dUTP-THP monocytes were treated with 1 μg/mL LPS for 3 hours and with 10 μM nigericin for an additional hour and characterized by nanoparticle tracking analysis. The trace represents particle concentration versus size distribution. (**F**) Western immunoblot of HERV-K dUTPase and the exosomal maker protein CD9 in EVs from GFP-THP or H.dUTP-THP; CD 9 is not detected in THP1 cell lysates because the membrane concentration of the exosomes is high on this blot. In (**C** and **D**), values are expressed as fold change compared with GFP-THP, and show *n* = 3, mean ± SEM. *P < 0.05, **P < 0.01, ***P < 0.001, ****P < 0.0001 versus GFP-THP; ^#^*P* < 0.05, ^##^*P* < 0.01, ^###^*P* < 0.001, ^####^*P* < 0.0001 versus H.dUTP-THP; and ^†^*P* < 0.05 versus H.dUTP-THP/a.H.dUTP Ab by a 1-way ANOVA followed by Tukey multiple comparison test.

Our previous studies localized elevated levels of HERV-K dUTPase to circulating monocytes and perivascular macrophages in PAH (14). We carried out coculture studies to determine whether monocytes secrete the HERV-K dUTPase protein to induce the gene expression changes in PAECs that we observed with the recombinant protein. PAECs were plated on the bottom of a 6-well dish and THP-1 monocytes transfected with HERV-K dUTPase or GFP vector as a control were added above a 1 μm Transwell filter. To assess the specific contribution of secreted HERV-K dUTPase from these monocytes, some PAEC cultures were pretreated with an anti–HERV-K dUTPase neutralizing antibody, or IgG isotype as a control, 1 hour before adding the monocytes (Figure 2A). We confirmed that THP-1 monocytes transfected with HERV-K dUTPase expressed high levels of *HERV-K dUTPase* mRNA throughout the 72 hours in coculture (Supplemental Figure 2A). After 24 hours, gene expression of *SNAIL* in PAECs was increased in the presence of THP-1 monocytes transfected with HERV-K dUTPase compared with GFP. However, *SLUG* was unchanged, as were the EC and SMC markers of EndMT at this early time point (Supplemental Figure 2B). However, following coculture for 72 hours, there was both a change in PAEC morphology to an elongated mesenchymal phenotype (Figure 2B), accompanied by an increase in *SNAIL*, *SLUG* and *ACTA2*, and a reduction in *CDH5* and *PECAM1* mRNA (Figure 2C). This response was ablated in PAECs pretreated with the HERV-K dUTPase neutralizing antibody but not with the control IgG isotype. In addition, HERV-K dUTPase released from monocytes cocultured with PAECs induced PAEC gene expression of *IL-6* and *VCAM1*, both previously implicated in PAH and with EndMT (22–24) (Figure 2D).

There is increasing evidence that monocytes communicate with ECs by transferring EVs (25, 26). HERV-K could have been directly secreted from monocytes and either bound to EVs or packaged in EVs if the HERV-K was exteriorized and susceptible to inhibition by the antibody. It is unlikely that HERV-K dUTPase exists in major excess in the secreted medium since we failed to detect it. To determine whether HERV-K dUTPase could be delivered to PAECs by EVs, we isolated similar amounts of EVs from media, in which HERV-K dUTPase and GFP-transfected THP-1 monocytes were cultured (Figure 2E), and documented abundant HERV-K dUTPase in EVs from HERV-K dUTPase-transfected THP-1 monocytes compared with those from GFP-transfected cells (Figure 2F).

### THP-1 HERV-K dUTPase EVs induce PH, EndMT, IL-6, and VCAM1.

**Figure 3:**
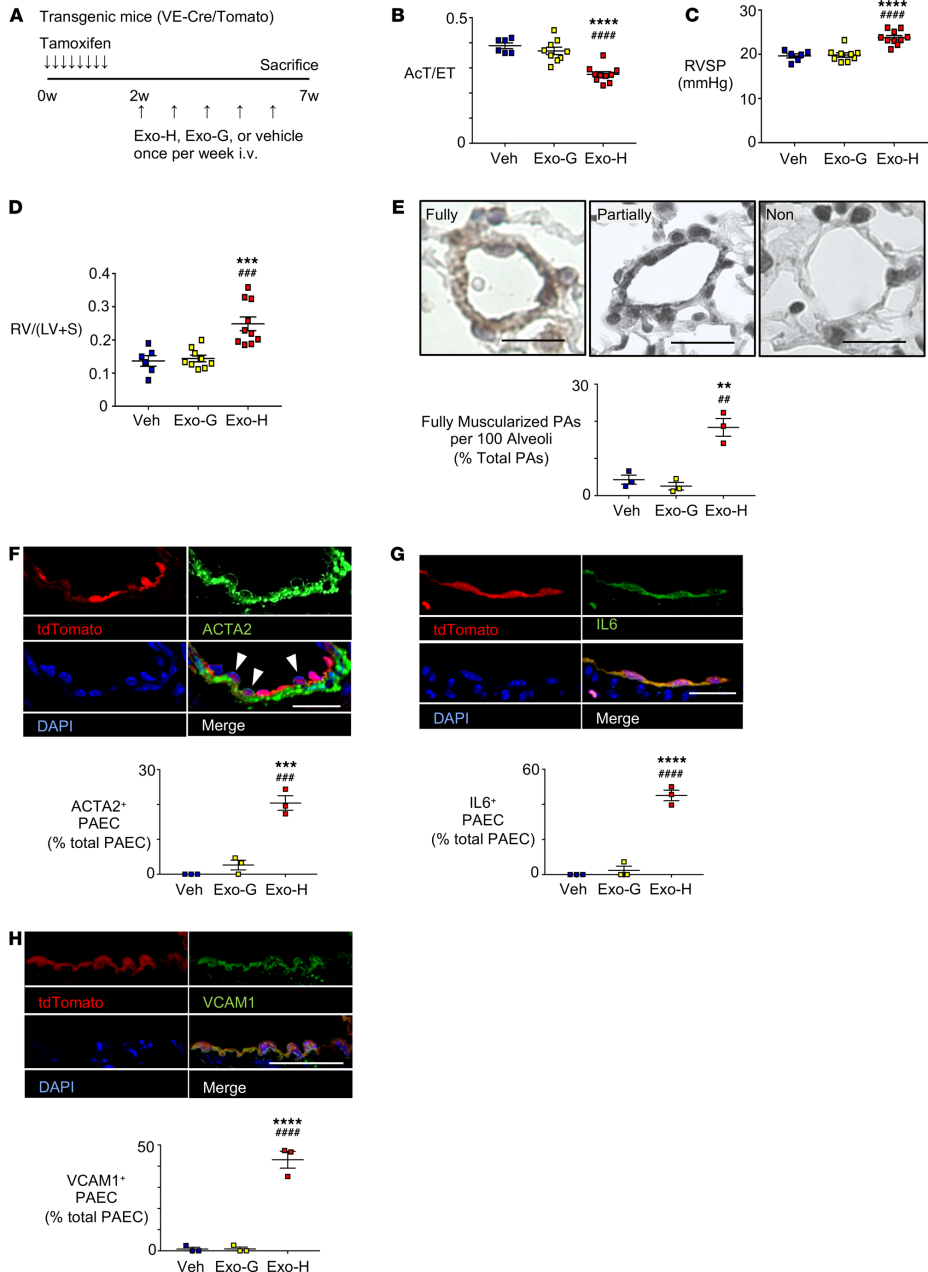
EVs containing HERV-K dUTPase derived from THP-1 monocytes induce PH, EndMT, and a proinflammatory phenotype in mice. (**A**) Schema of animal experiments. Adult male mice with an endothelial-specific inducible tdTomato cassette (VE-cadherin-CreER/tdTomato) (VE-Cre/Tomato) received 5 weekly tail-vein injections of EVs (10^9^ particles/g body weight) from culture medium of THP-1 monocytes overexpressing HERV-K dUTPase (Exo-H) (*n* = 9) or GFP (Exo-G) (*n* = 9). Control mice were injected with PBS vehicle (Veh) (*n* = 6). Mice treated with EVs containing HERV-K dUTPase versus GFP exhibited (**B**) decreased PA AcT per ejection time (AcT/ET), (**C**) increased RVSP, (**D**) increased RVH given by the ratio RV/(LV+S), and (**E**) an increase in the percent of fully muscularized distal arteries from 3 randomly selected mice per group. (**F**) Immunofluorescence microscopic images show ACTA2 (green) localized to tdTomato (red) positive PAECs (arrowhead) in PAs from endothelial-specific inducible tdTomato mouse. (Images for Exo-G shown in Supplemental Figure 3, E–G). Quantification below shows percent ACTA2 positive ECs of total ECs. Scale bar: 20 μm. (**G **and** H**) Images showing tdTomato (red) positive PAECs expressing IL-6 (green) (**G**) or VCAM1 (green) (**H**) in PAs from EC fate-mapped transgenic mouse, with quantification of percent IL-6 or VCAM1 positive ECs of total ECs. Scale bars: 20 μm. *n* = 3 mice in each condition; average of 10 PAs were evaluated per mouse, mean ±SEM. **P < 0.01, ***P < 0.001, ****P < 0.0001 versus Veh and ^##^*P* < 0.01, ^###^*P* < 0.001, ^####^*P* < 0.0001 versus Exo-G by 1-way ANOVA and Tukey multiple comparison test.****

We next assessed whether EVs derived from HERV-K dUTPase versus GFP-transfected monocytes could induce EndMT, proinflammatory PAEC, and pulmonary hypertension in mice as was seen in rats transfected with recombinant HERV-K dUTPase (14). Adult male VE-cadherin-CreER and tdTomato^fl/fl^ mice were treated with tamoxifen to fate map ECs (VE-cadherin-CreER/tdTomato) 1 week prior to receiving 5 once-weekly tail vein injections of EVs (10^9^ particles/g body weight) collected from medium in which THP-1 monocytes overexpressing HERV-K dUTPase or GFP were cultured (Figure 3A). There was no significant difference in body weight between mice treated with EVs from HERV-K dUTPase versus GFP-treated THP-1 cells and control mice injected with PBS vehicle (Supplemental Figure 3A). However, the mice treated with EVs containing HERV-K dUTPase developed pulmonary hypertension as judged by decreased pulmonary artery (PA) acceleration time (AcT) (Supplemental Figure 3B) and AcT per ejection time (Figure 3B), increased right ventricular systolic pressure (RVSP) (Figure 3C), right ventricular hypertrophy (RVH) (Figure 3D), and fully muscularized distal PAs (Figure 3E and Supplemental Figure 3, C and D). As evidence of EndMT, in mice treated with EVs containing HERV-K dUTPase, ACTA2 appeared in fate-mapped pulmonary arterial ECs in tissue sections (Figure 3F). We also observed heightened expression of IL-6 (Figure 3G) and VCAM1 in those cells (Figure 3H). Supplemental Figure 3, E–G, show the controls. These features are recapitulated in small vessels (Supplemental Figure 3, H–J). These studies, therefore, phenocopy both cell culture and animal experiments with HERV-K recombinant protein, suggesting that the HERV-K delivered by EVs could be responsible for the functional and morphological changes observed.

### HERV-K dUTPase induces SNAIL, IL-6, and VCAM1 via TLR4 and MCAM.

**Figure 4:**
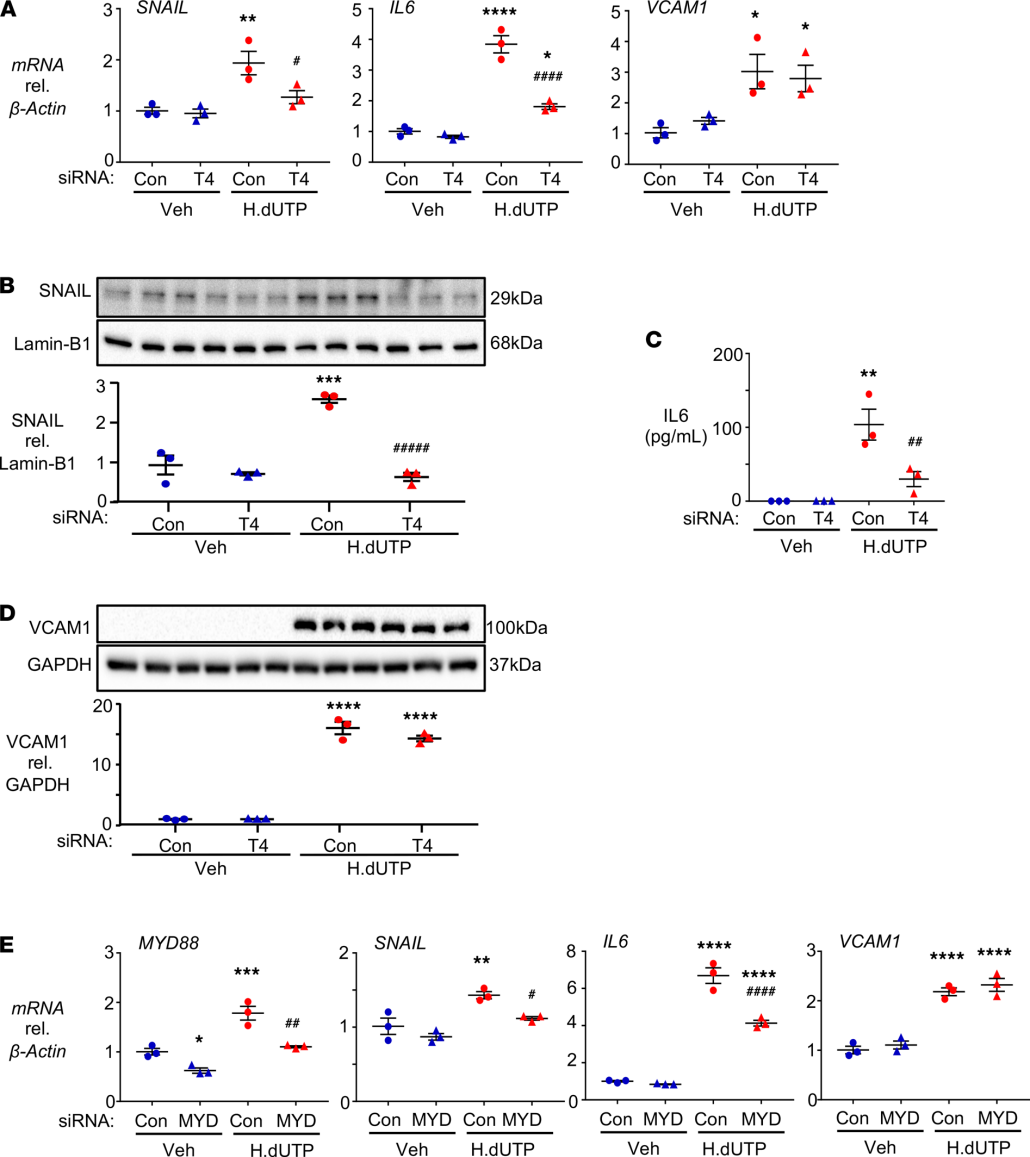
TLR4 and MYD88 mediate the expression of SNAIL and IL-6, but not VCAM1, in response to HERV-K dUTPase. (**A**–**D**) PAECs were transfected with siRNA targeting TLR4 (T4) or with nontargeting siRNA (Con) for 48 hours, then treated daily with 10 μg/mL HERV-K dUTPase (H.dUTP) or vehicle (Veh) for 72 hours. (**A**) *SNAIL*, *IL-6*, and *VCAM1* gene expression, assessed in whole cell lysates by qPCR. (**B**) SNAIL protein expression, assessed by immunoblot and densitometric quantification in nuclear extracts, using Lamin-B1 as the loading control. (**C**) Secreted IL-6, measured by ELISA in the EC media 24 hours following the addition of HERV-K dUTPase. (**D**) VCAM1 protein expression, assessed by immunoblot and densitometric quantification 72 hours after HERV-K dUTPase treatment in whole cell lysates using GAPDH as a loading control. (**E**) PAECs were transfected with siRNA targeting MYD88 (MYD) or with Con for 48 hours, then treated daily with 10 μg/mL HERV-K dUTPase (H.dUTP) or vehicle (Veh) for 72 hours. *SNAIL*, *IL-6*, and *VCAM1* gene expression changes were assessed by qPCR. For all panels, data are expressed as fold change compared with Veh/Con levels, showing the individual data points. *n* = 3 with mean ± SEM. *P < 0.05, **P < 0.01, ***P < 0.001, ****P < 0.0001 versus Veh/Con and ^#^*P* < 0.05, ^##^*P* < 0.01, ^####^*P* < 0.0001 versus H.dUTP/Con by 1-way ANOVA and Tukey multiple comparison test.

We next determined the mechanism by which recombinant HERV-K dUTPase delivered in EVs could serve as a ligand to induce EndMT via SNAIL, as well as IL-6, previously described in ECs in response to HERV-K dUTPase (14) and VCAM1, another proinflammatory molecule associated with EndMT (27, 28). Previous studies have shown that the EBV-encoded dUTPase contained in exosomes can engage TLR2 to activate NF-κB in dendritic cells (29). Several candidate receptors have been proposed as sensors of endogenous retroviruses (30); a member of the HERV-W family interacts with TLR4 to produce proinflammatory cytokines (15, 31), and recombinant HERV-K dUTPase induces NF-κB in a TLR2-dependent manner in the HEK293 cell line (16). Transfection of PAECs with TLR4 siRNA versus control siRNA effectively reduced *TLR4* mRNA (Supplemental Figure 4A) and TLR4 protein (Supplemental Figure 4B) and suppressed HERV-K dUTPase-mediated *SNAIL* as well as *IL-6,* but *VCAM1* mRNA was not reduced (Figure 4A). These results were replicated in PAECs treated with HERV-K dUTPase by the TLR4 neutralizing antibody but not IgG control (Supplemental Figure 4C). TLR4 siRNA, as opposed to control siRNA, similarly reduced SNAIL protein as determined by Western immunoblot (Figure 4B) and IL-6 levels assessed by ELISA (Figure 4C), but there was no significant impact on the induction of VCAM1 protein by HERV-K dUTPase (Figure 4D). Comparable results were obtained when we used siRNA to reduce the levels of an adaptor protein downstream of TLR4, i.e., myeloid differentiation primary response–88 (MYD88) (Figure 4E). The selectivity of the TLR4 response was evident in that reducing TLR2 by siRNA did not significantly alter the HERV-K dUTPase-mediated increase in *SNAIL* or *IL-6* mRNA (Supplemental Figure 4D) and TLR4 siRNA did not upregulate other receptors, such as TLR2 or TLR3 (Supplemental Figure 4E). We also determined that loss of BMPR2 did not result from HERV-K dUTPase (Supplemental Figure 4F), a feature previously shown to mediate EndMT in PAEC (4).

**Figure 5:**
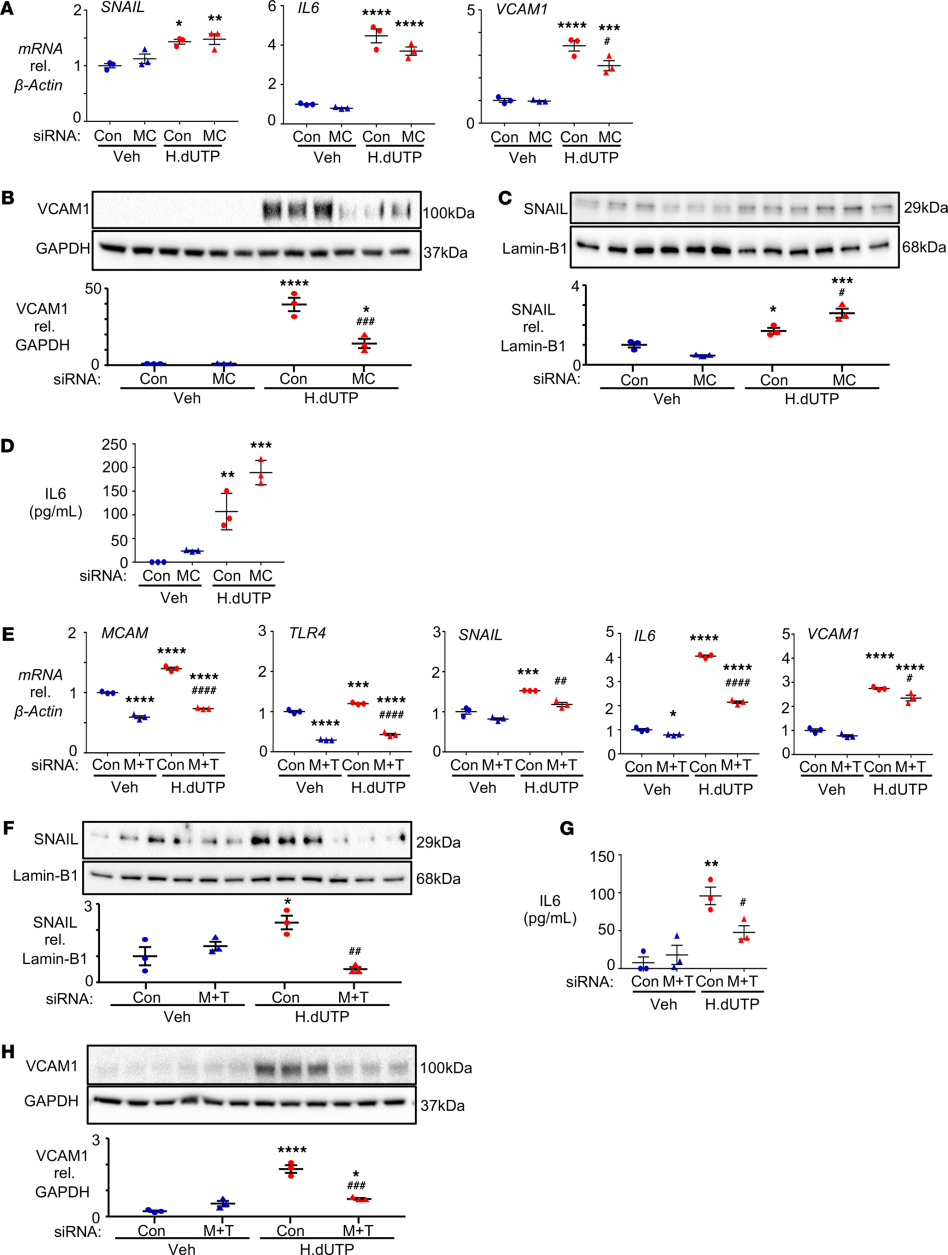
MCAM is required for the induction of VCAM1 by HERV-K dUTPase. (**A**–**D**) PAECs were transfected with siRNA targeting MCAM (MC) or with nontargeting siRNA (Con) for 48 hours, then treated as described for Figure 2. (**A**) *SNAIL*, *IL-6*, and *VCAM1* gene expression, assessed in whole cell lysates by qPCR. (**B**) VCAM1 protein expression was assessed in whole cell lysates by immunoblot and densitometric quantification 72 hours after HERV-K dUTPase treatment in whole cell lysates using GAPDH as a loading control. (**C**) SNAIL protein in nuclear extracts was assessed by immunoblot and densitometric quantification using Lamin-B as the loading control. (**D**) Secreted IL-6 was measured by ELISA in the EC media 24 hours following the addition of HERV-K dUTPase. (**E**–**H**) PAECs were transfected with siRNAs targeting MCAM and TLR4 (M+T) or with Con before HERV-K dUTPase treatment as described in **A**–**D**. (**E**) *MCAM*, *TLR4*, *SNAIL*, *IL-6*, and *VCAM1* mRNA were assessed as in **A**. (**F**) SNAIL protein expression was assessed by immunoblot and densitometric quantification, as in **B**. (**G**) Secreted IL-6 was measured by ELISA as in **C**. (**H**) VCAM1 protein expression was assessed by immunoblot and densitometric quantification as in **D**. For all panels, data are expressed as fold change compared with Veh/Con, and show *n* = 3, mean ± SEM. *P < 0.05, **P < 0.01, ***P < 0.001, ****P < 0.0001 versus Veh/Con and ^#^*P* < 0.05, ^##^*P* < 0.01, ^###^*P* < 0.001, ^####^*P* < 0.0001 versus H.dUTP/Con by a 1-way ANOVA followed by Tukey multiple comparison test.

The inability to suppress HERV-K dUTPase-mediated induction of *VCAM1* despite the loss of TLR4 or TLR2 led us to investigate other candidate receptors. These included CD36, a coreceptor for TLR4 and the TLR4/TLR6 heterodimer (32–35); TNFR1 and TNFR2, receptors for retrovirus productive proteins, e.g., HIV-1 (36); and IL1-receptor, since it had some homology to the cytoplasmic tail of TLRs (37). None of these candidates were required for HERV-K dUTPase induction of *VCAM1* as judged by siRNA experiments (data not shown). We therefore undertook affinity purification and mass spectrometry (APMS) to find PAEC cytoplasmic membrane proteins that directly interacted with HERV-K dUTPase (Supplemental Figure 5, A and B). From these APMS analyses, 3 HERV-K dUTPase interacting receptors were identified: neuropilin-1 (NRP1), endoglin (ENG), and MCAM. Of these, neither NRP1 nor ENG emerged as transducers of HERV-K dUTPase-mediated *VCAM1* mRNA as judged by reducing these receptors with siRNA (Supplemental Figure 5, C and D). However, when *MCAM* mRNA and protein levels were decreased by *MCAM* siRNA (Supplemental Figure 5, E and F), HERV-K dUTPase-mediated *VCAM1* mRNA (Figure 5A) and protein (Figure 5B) were reduced. No significant changes were observed with *MCAM* siRNA with respect to SNAIL (Figure 5C) or IL-6 (Figure 5D). As expected, reducing both *MCAM* and *TLR4* mRNA levels by siRNA resulted in the suppression of *SNAIL*, *IL-6*, and *VCAM1* mRNA (Figure 5E) and protein (Figure 5, F–H, respectively).

### HERV-K dUTPase induces SNAIL, VCAM1, and IL-6 via p38/NF-κB signaling.

**Figure 6:**
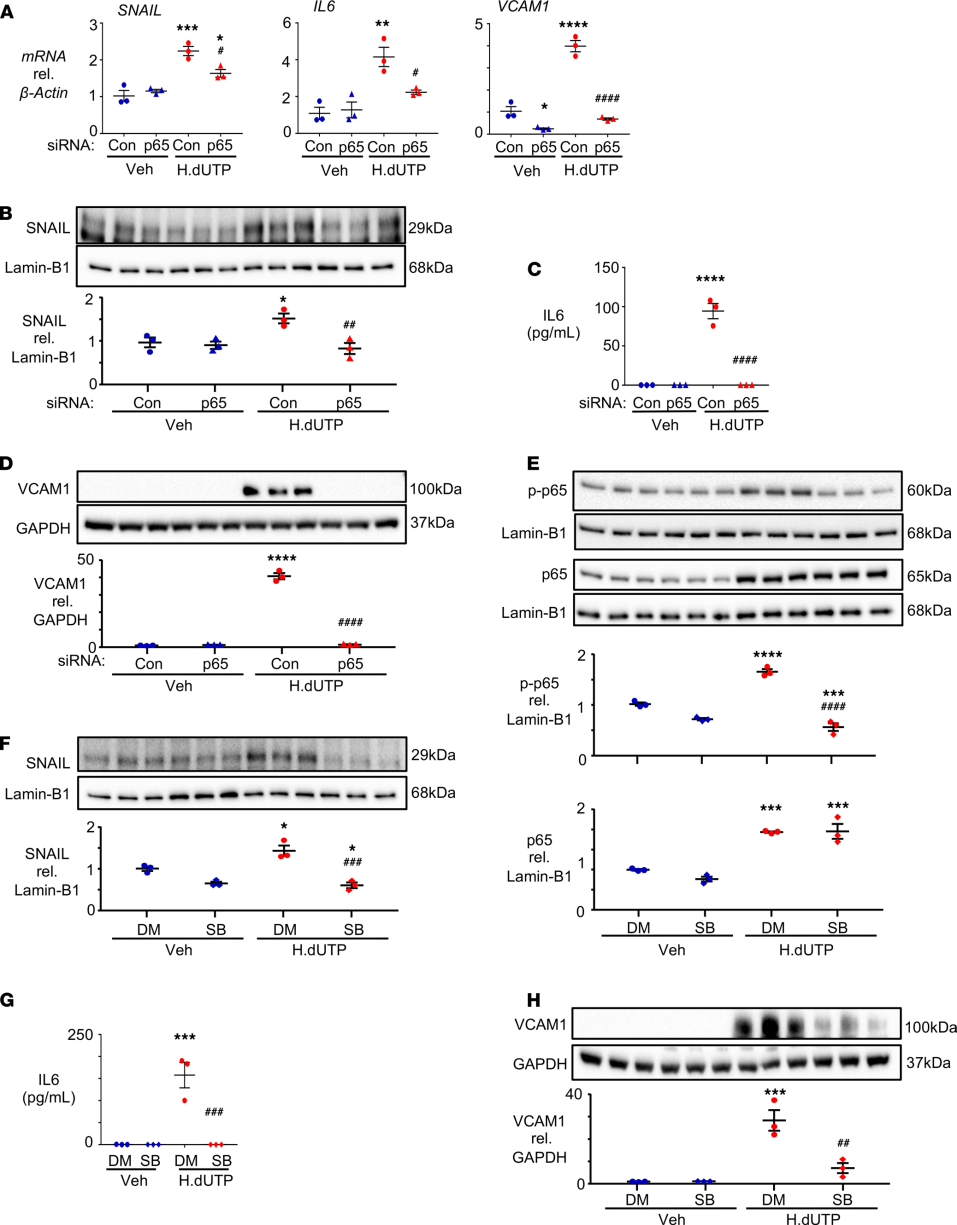
NF-κB and p-p38 are required for the induction of SNAIL, VCAM1, and IL-6 by HERV-K dUTPase. (**A**–**D**) PAECs were transfected with siRNA for p65 or nontargeting siRNA (Con), followed by treatment with 10 μg/mL of HERV-K dUTPase or vehicle as described for Figure 2. (**A**) *SNAIL, IL-6,* and *VCAM1* mRNA levels, assessed by qPCR. (**B**) Immunoblot and densitometric quantification of SNAIL protein expression. (**C**) Secreted IL-6, measured by ELISA in conditioned medium of PAECs following 24-hour treatment with HERV-K dUTPase. (**D**) Immunoblot and densitometric quantification of VCAM1 protein. (**E**–**H**) PAECs were pretreated with 10 μM of p38 inhibitor SB203580 (SB) or with the solvent DMSO (DM) for 2 hours, then treated with HERV-K dUTPase, as described above. (**E**) Immunoblot and densitometric quantification of p-p65 and p65 protein expression in nuclear extracts, assessed 1 hour after HERV-K dUTPase or vehicle treatment. (**F**) Immunoblot and densitometric quantification of SNAIL protein expression, assessed after daily HERV-K dUTPase or vehicle treatment as in **B**. (**G**) Secreted IL-6, measured by ELISA as in **C**. (**H**) Immunoblot and densitometric quantification of VCAM1 protein expression, assessed as described in **D**. Data are expressed as fold change compared with Veh/Con (**A**–**D**) or Veh/DM (**E**–**H**) and shown *n* = 3 with mean ± SEM. *P < 0.05, **P < 0.01, ***P < 0.001, ****P < 0.0001 versus Veh/Con (**A**–**D**) or Veh/DM (**E**–**H**) and ^#^*P* < 0.05, ^##^*P* < 0.01, ^###^*P* < 0.001, ^####^*P* < 0.0001 versus H.dUTP/Con or H.dUTP/DM, by a 1-way ANOVA followed by Tukey multiple comparison test.

*SNAIL* and many proinflammatory genes require NF-κB for their transcription (38, 39). Moreover, NF-κB is regulated by p38 MAPK, which is a downstream signal of TLR4 and MCAM (40, 41). Loss of *p65* mRNA and protein by siRNA (Supplemental Figure 6, A and B) suppressed HERV-K dUTPase-mediated induction of *SNAIL*, *IL-6*, and *VCAM1* mRNA (Figure 6A) and protein (Figure 6, B–D), suggesting that NF-κB is required for the expression of all 3 genes. We then determined that p38 MAPK regulates NF-κB activity. The p-p38 inhibitor SB203580 suppressed the phosphorylation of the NF-κB p65 subunit (Figure 6E) and inhibited HERV-K dUTPase induction of SNAIL, IL-6, and VCAM1 proteins (Figure 6, F–H).

### TLR4 and MCAM mediate p65 nuclear translocation and phosphorylation.

PAECs were transfected with siRNA for TLR4 (Supplemental Figure 7A) or MCAM (Supplemental Figure 7B) versus nontargeting siRNA to determine whether these receptors activated p38, JNK, and ERK signaling pathways in response to HERV-K dUTPase treatment. TLR4- and MCAM-mediated p38 phosphorylation was lost by siRNA reduction of either receptor. However, TLR4 also activated JNK MAPK and MCAM activated ERK MAPK. Using selective inhibitors of JNK and ERK, we were able to show that p-ERK “rescues” NF-κB nuclear translocation and phosphorylation when TLR4 and p-p38 are reduced by TLR4 siRNA (Supplemental Figure 7C) and p-JNK allows for NF-κB phosphorylation when MCAM and p-p38 are reduced by MCAM siRNA (Supplemental Figure 7D).

### SNAIL is induced by JNK/SMAD3, IL-6 by TLR4-STAT1, and VCAM1 by ERK/ATF2.

**Figure 7:**
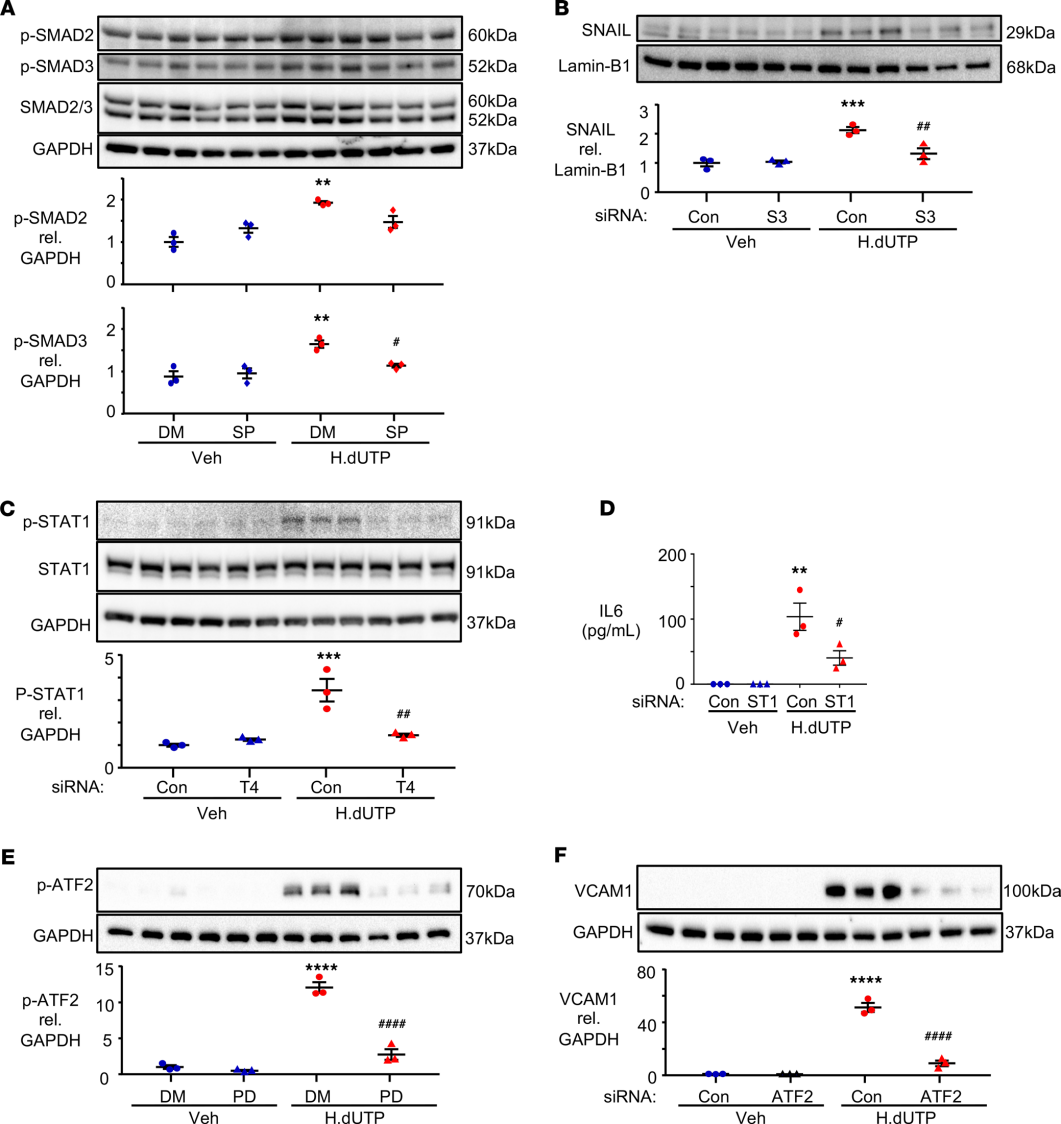
JNK/SMAD3 is required to induce SNAIL, TLR4-mediated STAT1 signaling is required for IL-6 induction, and ERK/ATF2 is required for the induction of VCAM1. (**A**) PAECs were pretreated with 30 μM of JNK inhibitor SP600125 (SP) or DMSO (DM) for 45 minutes, then treated with 10 μg/mL HERV-K dUTPase (H.dUTP) or vehicle (Veh) for 4 hours. p-SMAD2, p-SMAD3, and SMAD2/3 protein expression were assessed by immunoblot and densitometric quantification. (**B**) PAECs were transfected with siRNA targeting SMAD3 (S3) or with nontargeting siRNA (Con) for 48 hours, followed by HERV-K dUTPase treatment as described in Figure 2. SNAIL protein expression was assessed by immunoblot and densitometric quantification. (**C**) PAECs were transfected with siRNA targeting TLR4 (T4) or Con before HERV-K dUTPase treatment. p-STAT1 and STAT1 protein expression were assessed by immunoblot and densitometric quantification 4 hours after HERV-K dUTPase or vehicle treatment. (**D**) PAECs were transfected with siRNA targeting STAT1 (ST1) or with Con before HERV-K dUTPase treatment, and IL-6 was measured by ELISA in medium of treated PAECs as in Figure 2C. (**E**) PAECs were pretreated with 20 μM of ERK inhibitor PD98059 (PD) or DM for 2 hours, followed by HERV-K dUTPase or vehicle treatment for 4 hours. p-ATF2 protein expression was assessed by immunoblot and densitometric quantification. (**F**) PAECs were transfected with siRNA targeting ATF2 or with Con and treated with HERV-K dUTPase or vehicle as in Figure 2. VCAM1 protein was assessed by immunoblot and densitometric quantification at 72 hours. Data are expressed as fold change compared with Veh/DM (**A** and **E**) or Veh/Con (**B**–**D** and **F**) and show *n* = 3, mean ± SEM. **P < 0.01, ***P < 0.001, ****P < 0.0001 versus Veh/DM or Veh/Con and ^#^*P* < 0.05, ^##^*P* < 0.01, ^####^*P* < 0.0001 versus H.dUTP/DM or H.dUTP/Con by 1-way ANOVA and Tukey multiple comparison test.

While these studies indicated that NF-κB is required for transcription of *SNAIL*, *IL-6*, and *VCAM1*, they also revealed that it was not sufficient, so we assessed additional signaling molecules and transcription factors downstream of TLR4 and MCAM. As shown in Supplemental Figure 7A, HERV-K dUTPase induces the phosphorylation of JNK via TLR4. JNK and SMAD regulation of *SNAIL* in the process of epithelial-mesenchymal transition has been previously described (42, 43). Inhibition of JNK-suppressed phosphorylation of SMAD3 in response to HERV-K dUTPase (Figure 7A) and siRNA reduction of SMAD3 mRNA and protein (Supplemental Figure 8, A and B) reduced SNAIL protein expression (Figure 7B). It is not surprising that the TGF-β signaling pathway is involved in SNAIL-mediated EndMT (43). However, in other studies using TGF-β alone, the features of EndMT appear sooner, perhaps due to the culture conditions of our cells.

We next investigated whether HERV-K dUTPase induction of *IL-6* via TLR4 requires STAT1, a molecule previously related to TLR4-mediated stability of *IL-6* mRNA (44). Loss of TLR4 by siRNA suppressed the phosphorylation of STAT1 in response to HERV-K dUTPase (Figure 7C), and reducing *STAT1* mRNA and protein (Supplemental Figure 8, C and D) inhibited TLR4-mediated IL-6 (Figure 7D). As activating transcription factor-2 (ATF2) regulates *VCAM1* (45), we determined whether this occurred downstream of MCAM-mediated ERK signaling in response to HERV-K dUTPase (shown in Supplemental Figure 7B). Indeed, inhibition of ERK suppressed the phosphorylation of ATF2 in response to HERV-K dUTPase (Figure 7E), and reducing ATF2 mRNA and protein via siRNA (Supplemental Figure 8, E and F) resulted in a decrease in VCAM1 protein (Figure 7F).

## Discussion

Here we establish the underlying mechanism revealing how an elevation in HERV-K dUTPase in monocytes and macrophages (14) results in pulmonary hypertension. To address long-range signaling of HERV-K dUTPase from monocytes or macrophages, we show that this retroviral protein can be released in EVs to mediate gene expression in PAECs. Injection of monocyte EVs with elevated expression of HERV-K dUTPase is sufficient to induce pulmonary hypertension in mice related to EndMT and inflammation in the murine PAEC. We show how monocyte-derived HERV-K dUTPase engages 2 different PAEC receptors, causing elevated levels of specific signaling molecules and transcription factors that are required to produce an increase in IL-6, SNAIL, and consequent EndMT and the inflammatory cell adhesion molecule VCAM1. We show that TLR4 is required for expression of IL-6 and SNAIL and that MCAM is required for VCAM1, and that while both receptors activate p-p38 and NF-κB, different signaling molecules are required to induce coactivating transcription factors, i.e., p-STAT1 for *IL-6*, p-JNK–mediated p-SMAD3 for *SNAIL,* and p-ERK–mediated ATF2 for *VCAM1*. The studies do reinforce the need for novel therapies in PAH such as the use of TLR4 inhibitors currently in clinical trial for autoimmune disease (46). It could be tested in our mouse model if the usage of TLR4 in response to HERV-K dUTPase is similar.

As we and others have shown that EndMT is a feature of PAH (3, 4, 47), we first investigated whether HERV-K dUTPase secreted from monocytes might be an inducer of this pathological response. Previous studies have implicated the HERV-K envelope protein in epithelial-mesenchymal transition (48) and in breast cancer invasion and migration via activation of TGF-β and p-ERK (49). Our studies focused specifically on the dUTPase, since we previously showed that the HERV-K dUTPase can induce IL-6, and IL-6 has been implicated in EndMT in experimental pulmonary hypertension (18) and in epithelial-mesenchymal transition in cancer cells (50).

High levels of IL-6 have also been related to EndMT in response to loss of BMPR2 in experimental pulmonary hypertension (18, 51); however, BMPR2 levels were not significantly changed by HERV-K dUTPase. SNAIL, in addition to TWIST (52, 53), is the critical transcription factor involved in EndMT and PAH in human cells (4) and in experimental studies (18). While our study did not reveal a reduction in BMPR2 and the associated increase in HMGA1 (4), or an increase in TWIST in response to HERV-K dUTPase or in ZEB1, a transcription factor described in epithelial-mesenchymal transition (54), we cannot exclude the role of these transcription factors in concert with SNAIL. It is interesting that the elevation in SNAIL took place within 1 day after activation of PAEC by HERV-K dUTPase, followed first by a reduction in PECAM1 and CDH5 and later by an increase in SM22 and ACTA2. This suggests that endothelial changes and loss of cell-cell contact may be required for upregulation of mesenchymal genes.

Blocking TLR4 could not suppress the induction of another proinflammatory molecule, VCAM1, suggesting a separate receptor interacting with HERV-K dUTPase. This was surprising, because in multiple sclerosis a functionally similar molecule, ICAM, is upregulated by HERV-W interaction with TLR4 (55). Of the 3 receptors identified that could function as regulators of gene expression, MCAM, identified first as a component of EC-EC interaction (56), appeared to be the regulator of VCAM1. Although previously described as coexpressed with VCAM1 (57), there has been no previous study, to our knowledge, linking MCAM with endogenous retroviral protein interaction or with VCAM1. It is of further interest that the immunoprecipitation of HERV-K dUTPase did not reveal an interaction with TLR4. This may be due to the conditions of the assay, particularly if the interaction was of lower affinity than with MCAM.

In searching for a transcription factor downstream of TLR4 and MCAM signaling that would be essential for HERV-K–mediated gene regulation, we first investigated NF-κB. Our previous study showed that HERV-K induces SAM domain- and HD domain-containing protein-1 (14) and HIV induction of SAMHD1 requires TLR4-mediated NF-κB (58). p-p38 is a consequence of HERV-K dUTPase interaction with both TLR4 and MCAM and is required to induce transcription of all 3 genes—SNAIL, IL-6, and VCAM1—because of its role in the activation of NF-κB (40). Although p-JNK (59) and p-ERK (60) are also associated with NF-κB activation, our data show that these MAPKs are alternative activators when p-p38 is disabled, findings not, to our knowledge, previously reported. Our model of HERV-K–mediated gene regulation suggests a unified endothelial response that attracts inflammatory cells both via upregulation of a powerful cytokine, IL-6, and an adhesion molecule, VCAM1. The switch to a mesenchymal phenotype might serve to protect ECs from apoptosis, as has been shown experimentally (18) and in this study.

The transfer of HIV and EBV viral particles via exosomes from dendritic cells to T cells was previously described (61). Later studies have shown that monocytes and macrophages incorporate HIV as well as other proteins in exosome transfer that ensure high replication efficiency of the virus (62). It would be of interest to determine whether, in addition to HERV-K, signaling molecules and microRNAs are present in the EVs (63) that could also impact the changes observed. It has been shown that exosomes containing HIV reorganize TLR4 in lipid rafts in macrophages (64) and alter cholesterol biosynthesis. It is possible that this mechanism is also required for HERV-K dUTPase in EVs to engage TLR4 and MCAM. Previously, it has been shown that exosomes from HIV-infected monocytes enter ECs and induce adhesion molecules (ICAM) and cytokines such as IL-6 via activation of NF-κB and TLR4 (26). However, the role of monocyte-derived exosomes containing endogenous viral proteins in inducing EndMT has not, to our knowledge, been described. We also do not know whether the HERV-K dUTPase is on the membrane surface of the EVs or internalized.

Our study suggests that the long-range transfer of HERV-K dUTPase can both transform cells and ignite an inflammatory response. If there is transfer of HERV-K dUTPase RNA in EVs, then the production of dsRNA can lead to a chronic interferon response (65, 66), as was observed with the dUTPase RNA produced by AAV9 (67). Previous studies have shown that interferon levels are elevated in PAH (68) and chronic interferon therapy can induce PAH (69). Our report links activation of an endogenous retroviral protein in monocytes with the pathogenesis of PAH through specific receptors and transcription factors that could be therapeutically targeted to prevent and potentially reverse progressive disease.

## Methods

### HERV-K dUTPase protein purification.

Recombinant HERV-K dUTPase protein was provided by The Ohio State University Wexner Medical Center. The HERV-K gene encoding the dUTPase was cloned into the pTrcHis Topo TA expression vector, and the sequence was verified by DNA sequencing analysis as previously described (16). The purity of the protein was assessed by SDS-PAGE and capillary-liquid chromatography nanospray tandem mass spectrometry performed at the Ohio State University Mass Spectrometry and Proteomics Facility. High-purity dUTPase preparations, free of contaminating DNA, RNA, lipopolysaccharide, and peptidoglycan, were used.

### PAEC culture and recombinant HERV-K dUTPase treatment.

Commercial human PAECs (PromoCell) were cultured in EC media supplemented with 5% FBS, EC growth supplement, and penicillin (50 U/mL)/streptomycin (50 U/mL) and used at passage 3–8. All tissue culture reagents were purchased from ScienCell. For experiments assessing the effect of recombinant HERV-K dUTPase, human PAECs were treated with 10 μg/mL of recombinant HERV-K dUTPase once a day for 3 days, followed by every 3 days up to 20 days. PAECs were evaluated for alterations in cell phenotype, gene, and protein expression at 1, 3, 10, and 20 days after initial treatment using quantitative real-time PCR (qPCR), immunoblotting, and immunofluorescence staining using a confocal microscope (FV1000, Olympus). Apoptosis was assessed on day 1, 10, and 20 following overnight (16 hours) serum withdrawal. Cells were then incubated for 1 hour in 30 μL of Caspase 3/7 Luciferase Reagent Mix (Promega), and total luminescence was measured in a plate reader (BioTek Synergy H1 Hybrid Reader).

### Immunofluorescence.

Lung sections were from lungs harvested from patients undergoing lung transplantation for PAH as well as from unused donor lungs as controls, obtained through the Pulmonary Hypertension Breakthrough Initiative (PHBI) Network. The lungs were procured at the Transplant Procurement Centers and de-identified patient data were obtained via the Data Coordinating Center at the University of Michigan. Clinicopathological baseline characteristics of patients and donors are given in Supplemental Tables 1 and 2.

Formaldehyde-fixed, paraffin-embedded human or mouse lung tissue sections were deparaffinized and rehydrated. Sections were reacted with 0.3% hydrogen peroxide to block endogenous peroxidase and with 1% BSA in PBS to block nonspecific staining (70). Then, the sections were incubated with primary antibodies against ACTA2, CDH5, SNAIL/SLUG, vWF, IL-6, or VCAM1 (details below) overnight at 4°C. Following overnight incubation, sections were washed 3 times with PBS and incubated with Alexa Fluor 488 (labeled anti-mouse or rabbit, 1:400) or Alexa Fluor 594 (labeled anti-mouse or rabbit, 1:400) conjugated secondary antibody (Invitrogen) for 1 hour at room temperature. Sections were washed 3 times with PBS, mounted with mounting medium containing DAPI (Vector Laboratories), and stored at 4°C until analysis. Confocal analysis was performed using a confocal laser-scanning microscope (FV1000, Olympus).

Antibodies used for immunohistochemistry: ACTA2, 1:200 (Sigma-Aldrich, catalog A2547); CDH5, 1:200 (Abcam, catalog 33168); SNAIL/SLUG, 1:200 (Abcam, catalog 85936); vWF, 1:400 (Abcam, catalog 6994); IL-6 1:200 (Abcam, catalog 208113); and VCAM1, 1:250 (Abcam, catalog 134047).

### Nuclear, cytoplasmic, and membrane fractions.

To assess the nuclear expression of SNAIL and p65, nuclear fractions of PAECs were isolated using NE-PER Nuclear Extraction Reagents (Thermo Fisher Scientific). To assess the affinity of membrane proteins for HERV-K dUTPase, membrane fractions of PAECs were isolated using Subcellular Protein Fractionation Kit for Cultured Cells (Thermo Fisher Scientific) according to the manufacturer’s instruction.

### qPCR.

Total RNA was extracted and purified from cells using the Quick-RNA MiniPrep Kit (Zymo Research). The quantity and quality of RNA was determined by using a spectrophotometer. A total of 2 μg of RNA was used as a template for reverse-transcription PCR with the High Capacity RNA to cDNA Kit (Applied Biosystems) according to the manufacturer’s instructions. qPCR was performed using a pair of 1 μL of 5 μM primers, 5 μL of PowerUp SYBR Green PCR Master Mix (Applied Biosystems), 2 μL of dH_2_O, and 2 μL of 1 ng cDNA sample in a 10 μL reaction. Each measurement was carried out in triplicate using a CFX384 Real-Time System (Bio-Rad). The PCR conditions were 95°C for 2 minutes, followed by 40 cycles of 95°C for 15 seconds, and 60°C for 60 seconds. Primer sequences were designed using PrimerBank and are listed below. The gene expression levels were normalized to the expression level of β-Actin. Primers used for qPCR are listed in Supplemental Table 3.

### Western immunoblotting.

Cells were trypsinized and washed with cold PBS, then lysed in lysis buffer (10 mM Tris-HCl and 1% SDS) and boiled at 95°C for 5 minutes. After centrifugation at 20,000*g* for 20 minutes at 4°C, the supernatant was collected. Protein concentration was determined by the BCA assay (Thermo Fisher Scientific). Equal amounts of protein were separated by SDS-PAGE and transferred to a polyvinylidene difluoride membrane. After blocking with 5% milk in 0.1% Tween-TBS (Promega), the membranes were incubated in 5% BSA with primary antibodies as listed below. Then, appropriate secondary antibodies were used. Normalization for total protein was carried out by reprobing the membrane with an antibody against β-Actin, GAPDH, histone 3, or Lamin-B1. Signal was detected by West Femto Substrate (Thermo Fisher Scientific) and Bio-Rad ChemiDoc XRS system. Densitometric quantifications were performed using ImageJ software (NIH). Antibodies used for Western immunoblotting are listed in Supplemental Table 4.

### siRNA transfection.

ON TARGETplus SMARTpool siRNA (Horizon), Silencer Select siRNA (Thermo Fisher Scientific), and Gene Silencer (Santa Cruz Biotechnology) (see list below) were transfected into PAECs by the 4D Nucleofector Core unit using p5 Primary Cell Solution according to manufacturer’s recommendations (Lonza). The knockdown efficiency was determined by qPCR and immunoblotting. PAECs transfected with siRNA were incubated for 48 hours, and then treated with 10 μg/mL of HERV-K dUTPase for 72 hours.

siRNAs used for transfection: TLR4 (Ambion, catalog s14194); TLR2 (Ambion, catalog s162); TLR6 (Ambion, catalog s20215); MYD88 (Ambion, catalog s9134); NRP1 (Dharmacon, catalog L019484); ENG (Dharmacon, catalog L011026); MCAM (Ambion, catalog s8572); RELA (Ambion, catalog s11915); SMAD3 (Ambion, catalog s535079); STAT1 (Ambion, catalog s279); ATF2 (Santa Cruz, catalog sc29205); Negative Control No.1 (Ambion, catalog 4390844); Nontargeting Control (Dharmacon, catalog D001810); and Control siRNA-A (Santa Cruz, catalog Sc37007).

### ELISA.

In PAECs treated with HERV-K dUTPase, the IL-6 protein was measured using the Quantikine ELISA kit for human IL-6 (R&D Systems) according to the manufacturer’s protocol.

### Affinity purification followed by mass spectrometry (APMS).

AP was performed using the Pierce Pull-Down PolyHis Protein: Protein Interaction Kit (Thermo Fisher Scientific) according to manufacturer’s instructions. Briefly, the previously purified 150 μg of polyhistidine-tagged HERV-K dUTPase protein (provided by The Ohio State University Wexner Medical Center) was immobilized on a cobalt-chelate resin column. Then, in order to capture the prey protein, the membrane protein isolated from 0.2 g wet weight of human PAECs was added to the prepared column, followed by the incubation at 4°C for 2 hours with gentle rocking motion on a rotating platform. To elute bait-prey protein, 290 mM imidazole elution buffer: 1:1 of TBS: Pierce Lysis Buffer (Thermo Fisher Scientific) and imidazole, were added to the spin column, followed by incubation for 5 minutes of gentle rocking on a rotating platform and centrifugation at 1250*g* for 30 seconds. For MS analysis, samples were reduced with 5 mM DTT in 120 μL of 50 mM ammonium bicarbonate. Following reduction, proteins were alkylated using 10 mM acrylamide for 30 minutes at room temperature to cap cysteines. Digestion was performed using Trypsin/LysC (Promega) overnight at 37°C. Following digestion and acid quenching, samples were passed over HILIC resin (Resyn Biosciences) and dried in a speed vac and then reconstituted in 10 μL reconstitution buffer (2% acetonitrile with 0.1% formic acid); 3 μL of the reconstituted peptides were injected on the instrument. All MS experiments were performed using an Orbitrap Fusion Tribrid mass spectrometer (Thermo Fisher Scientific) with an attach Acquity M-Class UPLC (Waters Corporation) liquid chromatograph. A pulled-and-packed fused silica C18 reverse phase column containing 1.8-micron C18 beads from Dr. Maisch (Ammerbuch-Entringen, Germany) and a length of approximately 25 cm was used over an 80 minute gradient. A flow rate of 300 nL/min was used with the mobile phase A consisting of aqueous 0.2% formic acid and mobile phase B consisting of 0.2% formic acid in acetonitrile. Peptides were directly injected onto the analytical column. The mass spectrometer was operated in a data dependent fashion, with MS1 survey spectra collected in the orbitrap and MS2 fragmentation using CID in the ion trap. For data analysis, the RAW data files were processed using Byonic v2.14.27 (Protein Metrics) to identify peptides and infer proteins against the human UniProt database containing isoforms concatenated with synthesized sequences. Proteolysis was assumed to be tryptic in nature and allowed for ragged n-terminal digestion and up to 2 missed cleavage sites. Precursor mass accuracies were held within 12 ppm, with MS/MS fragments held to a 0.4 Da mass accuracy. Proteins were held to a false discovery rate of 1%, using standard approaches (71). Our data are now uploaded and are in the public domain:https://massive.ucsd.edu/ProteoSAFe/dataset.jsp?task=e40d965b00e64357b1bfa45e414e758e

### THP-1 monocyte culture and transfection.

THP-1 monocytes (ATCC TIB-202) were cultured in RPMI1640 media (Gibco) supplemented with 10% FBS, 0.05 nM β-mercaptoethanol, and penicillin (50 U/mL)/streptomycin (50 U/mL). To generate a stable HERV-K dUTPase overexpressing THP-1 cell line, TransIT-2020 Transfection Reagent (Mirus Bio) was used with the following DNA vectors: pPB-HERV-KdUTPase-mCherry/Puro-EF1A, pRP-hyPBase-mCherry-CAG, and pPB-EGFP-mCherry/Puro-EF1A (VectorBuilder). In brief, a total of 19 μg of DNA vectors (3 μg of pRP-hyPBase with 16 μg of pPB-HERV-KdUTPase or 3 μg of pRP-hyPBase with 16 μg of pPB-EGFP) and 57 μL of TransIT-2020 reagent were mixed in 1.9 mL Opti-MEM (Gibco). Then, this solution was incubated for 20 minutes at room temperature and added to 5 *×* 10^5^ cells/mL THP-1 monocytes cultured in 19.7 mL complete THP-1 growth media for transfection. After 48 hours of transfection, THP-1 monocytes were collected, and then fresh media were added to the cells. The transfected cells were allowed to proliferate for 48 hours, and then the cell culture medium was replaced with fresh medium plus 0.75 μg/mL of puromycin in order to select stable clones expressing HERV-K dUTPase. After 48 hours of puromycin selection, THP-1 monocytes were collected, and then fresh media were added. After 3–4 weeks, the cells were collected and examined for *HERV-K dUTPase* mRNA expression by qPCR. Selected clones were used for further experiments.

### Coculture experiments.

Commercial human PAECs were plated onto a 6-well plate (2.0 *×* 10^5^ cells/well) and cultured in complete EC media. After 24 hours, HERV-K dUTPase or GFP-transfected THP-1 monocytes (1 *×* 10^6^ cells/well) were seeded on the Transwell membrane inserts (1 μm pore size, BD Falcon, catalog 353102) and cocultured with PAECs in complete EC media for 3 days. For experiments assessing the effect of HERV-K dUTPase released from the THP-1 monocytes, PAECs were pretreated with anti–HERV-K dUTPase antibody or IgG isotype 1 hour before starting coculture. Following coculture for 24 and 72 hours, PAECs were evaluated for alterations in cell phenotype and gene expression using microscopy and qPCR, respectively, and THP-1 monocytes were evaluated for *HERV-K dUTPase* expression by qPCR.

### Isolation and characterization of EVs.

After 24-hour culture of 1 *×* 10^6^ cells/mL HERV-K dUTPase or GFP overexpressing THP-1 monocytes in 30 mL RPMI1640 media with exosome-depleted 10% FBS (Gibco), THP-1 monocytes were primed with 1 μg/mL LPS (Sigma-Aldrich) for 3 hours and treated with 10 μM nigericin (Sigma-Aldrich) for an additional 1 hour to stimulate the release of EVs. After that, the culture media was collected and precleared by centrifugation at 300*g* for 10 minutes, then at 2000*g* for 10 minutes to eliminate dead cells and cellular debris. The supernatant was then ultra-centrifuged at 100,000*g* for 70 minutes at 4°C (Sorvall WX+ Ultracentrifuge, Thermo Fisher Scientific), followed by washing of the EV pellet with PBS at 100,000*g* for 70 minutes at 4°C to remove LPS and nigericin. To characterize the collected EVs, pellets were resuspended in saline, and the number and size of the particles measured by NanoSight (Malvern Panalytical). The concentration of the particles (particles/mL) was used to determine the volume of the injected suspension to achieve the desired dosing (particles/g BW) in the mice. For western blot analysis, we normalized for EV quantity based on the protein content of the EVs, determined using Pierce microplate BCA protein assay kit (Thermo Fisher Scientific).

### Mouse model for the induction of pulmonary hypertension, EndMT, and proinflammatory phenotype by EVs derived from HERV-K dUTPase overexpressed THP-1 monocytes.

To monitor EndMT, mice with an endothelial-specific inducible tdTomato were created by breeding VE-cadherin-CreER and tdTomato^fl/fl^ mice (VE-cadherin-CreER/tdTomato) in our laboratory. The tdTomato transgene was expressed in ECs by administering 2 mg tamoxifen per day i.p. for 8 days when the mice were 8–12 weeks of age. Male transgenic mice were randomly assigned to a treatment or control group, controlling for age. Male mice were used, as previously the response to the recombinant protein HERV-K dUTPase was carried out in male rats (14). The number of mice per experiment is indicated in the figure legends. A total of 1 *×* 10^9^ particles/g of EVs isolated from the medium, in which THP-1 monocytes overexpressing HERV-K dUTPase or GFP were cultured, were i.v. injected into male transgenic mice on days 0, 7, 14, 21, and 28 after completing the tamoxifen treatment. A second control group of male transgenic mice were treated with PBS instead of EVs. Thirty-five days after the first injection of EVs, RVSP, RVH, cardiac function and output, as well as PA AcT, were all measured according to methods previously published by our group (72). The heart and lungs were perfused with PBS, the left lung fixed, and sections embedded in paraffin for immunohistochemistry and immunofluorescence. The right lung was snap-frozen in liquid N_2_ and kept at –80°C.

### Quantification of distal PA muscularization.

To assess muscularization of distal PA, immunohistochemistry for ACTA2 was performed. The percentage of fully, partially, and nonmuscular arteries at alveolar duct and wall level were quantified per field of 100 alveoli in 4 fields in each mouse.

### Quantification of tdTomato-positive ECs expressing ACTA2, IL-6, and VCAM1.

To assess ACTA2, IL-6, and VCAM1 expressing tdTomato-positive ECs, immunofluorescence staining for ACTA2, IL-6, and VCAM1 was performed. The percentages of ACTA2, IL-6, and VCAM1 expressing tdTomato-positive ECs were quantified relative to total ECs of 10 PAs in each mouse.

### Statistics.

All data represent mean ± SEM. Multiple group comparisons were calculated using a 1-way ANOVA followed by Tukey multiple comparison test; 2-sided unpaired Student’s *t* test analyses were used for comparison of 2 groups. A *P* value of less than 0.05 was considered significant. The number of experiments, the animals per group, and the statistical test used are indicated in the figure legends or in the appropriate text. Statistical analysis was performed using GraphPad Prism 7.

### Study approval.

The Animal Care Committee of Stanford University approved all protocols, in keeping with the regulations of the American Physiological Society. Procurement of the tissues from human subjects is approved by the Administrative Panels on Human Subjects in Medical Research at the Pulmonary Hypertension Breakthrough Initiative Transplant Procurement Centers.

## Author contributions

SO conceived and performed the experiments, interpreted data and wrote the manuscript. TS, ST, JRM, DPM, RLH, AC, DL, and LW contributed to conception and design, acquisition of data, and analysis and interpretation of data. MEA provided the recombinant HERV-K dUTPase protein and anti–HERV-K dUTPase antibody and edited the manuscript. MR oversaw study design, data acquisition and analysis, and manuscript preparation and editing.

## Supplementary Material

Supplemental data

## Version 1 06/29/2021

In-Press Preview

## Version 2 08/09/2021

Electronic publication

## References

[1] Rabinovitch M (2012). Molecular pathogenesis of pulmonary arterial hypertension. J Clin Invest.

[2] Arciniegas E (2007). Perspectives on endothelial-to-mesenchymal transition: potential contribution to vascular remodeling in chronic pulmonary hypertension. Am J Physiol Lung Cell Mol Physiol.

[3] Ranchoux B (2015). Endothelial-to-mesenchymal transition in pulmonary hypertension. Circulation.

[4] Hopper RK (2016). In pulmonary arterial hypertension, reduced BMPR2 promotes endothelial-to-mesenchymal transition via HMGA1 and its target slug. Circulation.

[5] Alastalo TP (2011). Disruption of PPARγ/β-catenin-mediated regulation of apelin impairs BMP-induced mouse and human pulmonary arterial EC survival. J Clin Invest.

[6] Machado RD (2001). BMPR2 haploinsufficiency as the inherited molecular mechanism for primary pulmonary hypertension. Am J Hum Genet.

[7] Xie Y (2012). Impaired cardiac microvascular endothelial cells function induced by Coxsackievirus B3 infection and its potential role in cardiac fibrosis. Virus Res.

[8] Hughes JF, Coffin JM (2001). Evidence for genomic rearrangements mediated by human endogenous retroviruses during primate evolution. Nat Genet.

[9] Grow EJ (2015). Intrinsic retroviral reactivation in human preimplantation embryos and pluripotent cells. Nature.

[10] Deniz O (2020). Endogenous retroviruses are a source of enhancers with oncogenic potential in acute myeloid leukaemia. Nat Commun.

[11] Foerster J (2005). Haplotype sharing analysis identifies a retroviral dUTPase as candidate susceptibility gene for psoriasis. J Invest Dermatol.

[12] Groger V, Cynis H (2018). Human endogenous retroviruses and their putative role in the development of autoimmune disorders such as multiple sclerosis. Front Microbiol.

[13] Staege MS, Emmer A (2018). Editorial: endogenous viral elements-links between autoimmunity and cancer?. Front Microbiol.

[14] Saito T (2017). Upregulation of human endogenous retrovirus-K is linked to immunity and inflammation in pulmonary arterial hypertension. Circulation.

[15] Rolland A (2006). The envelope protein of a human endogenous retrovirus-W family activates innate immunity through CD14/TLR4 and promotes Th1-like responses. J Immunol.

[16] Ariza ME, Williams MV (2011). A human endogenous retrovirus K dUTPase triggers a TH1, TH17 cytokine response: does it have a role in psoriasis?. J Invest Dermatol.

[17] Rabinovitch M (2014). Inflammation and immunity in the pathogenesis of pulmonary arterial hypertension. Circ Res.

[18] Tian W (2019). Phenotypically silent bone morphogenetic protein receptor 2 mutations predispose rats to inflammation-induced pulmonary arterial hypertension by enhancing the risk for neointimal transformation. Circulation.

[19] Diez M (2010). Endothelial progenitor cells undergo an endothelial-to-mesenchymal transition-like process mediated by TGFbetaRI. Cardiovasc Res.

[20] Van der Feen DE (2019). Multicenter preclinical validation of BET inhibition for the treatment of pulmonary arterial hypertension. Am J Respir Crit Care Med.

[21] Tang H (2018). Endothelial HIF-2α contributes to severe pulmonary hypertension due to endothelial-to-mesenchymal transition. Am J Physiol Lung Cell Mol Physiol.

[22] Zhong T (2020). Adaptation of endothelial cells to shear stress under atheroprone conditions by modulating internalization of vascular endothelial cadherin and vinculin. Ann Transl Med.

[23] Lai B (2018). Atheroprone flow enhances the endothelial-to-mesenchymal transition. Am J Physiol Heart Circ Physiol.

[24] Le Hiress M (2015). Proinflammatory signature of the dysfunctional endothelium in pulmonary hypertension. Role of the macrophage migration inhibitory factor/CD74 complex. Am J Respir Crit Care Med.

[25] Sáez T (2019). Exosomes derived from monocytes and from endothelial cells mediate monocyte and endothelial cell activation under high d-glucose conditions. Immunobiology.

[26] Tang N (2016). Monocyte exosomes induce adhesion molecules and cytokines via activation of NF-κB in endothelial cells. FASEB J.

[27] Murdoch CE (2014). Endothelial NADPH oxidase-2 promotes interstitial cardiac fibrosis and diastolic dysfunction through proinflammatory effects and endothelial-mesenchymal transition. J Am Coll Cardiol.

[28] Kovacic JC (2019). Endothelial to mesenchymal transition in cardiovascular disease: JACC state-of-the-art review. J Am Coll Cardiol.

[29] Ariza ME (2013). Epstein-Barr virus encoded dUTPase containing exosomes modulate innate and adaptive immune responses in human dendritic cells and peripheral blood mononuclear cells. PLoS One.

[30] Hurst TP, Magiorkinis G (2015). Activation of the innate immune response by endogenous retroviruses. J Gen Virol.

[31] Rolland A (2005). Correlation between disease severity and in vitro cytokine production mediated by MSRV (multiple sclerosis associated retroviral element) envelope protein in patients with multiple sclerosis. J Neuroimmunol.

[32] Stewart CR (2010). CD36 ligands promote sterile inflammation through assembly of a Toll-like receptor 4 and 6 heterodimer. Nat Immunol.

[33] Chavez-Sanchez L (2014). The role of TLR2, TLR4 and CD36 in macrophage activation and foam cell formation in response to oxLDL in humans. Hum Immunol.

[34] Kiyan Y (2014). oxLDL induces inflammatory responses in vascular smooth muscle cells via urokinase receptor association with CD36 and TLR4. J Mol Cell Cardiol.

[35] Cao D (2016). CD36 regulates lipopolysaccharide-induced signaling pathways and mediates the internalization of Escherichia coli in cooperation with TLR4 in goat mammary gland epithelial cells. Sci Rep.

[36] Pasquereau S (2017). Targeting TNF and TNF receptor pathway in HIV-1 infection: from immune activation to viral reservoirs. Viruses.

[37] Akira S, Takeda K (2004). Toll-like receptor signalling. Nat Rev Immunol.

[38] Barbera MJ (2004). Regulation of snail transcription during epithelial to mesenchymal transition of tumor cells. Oncogene.

[39] Julien S (2007). Activation of NF-kappaB by Akt upregulates Snail expression and induces epithelium mesenchyme transition. Oncogene.

[40] Olson CM (2007). p38 mitogen-activated protein kinase controls NF-kappaB transcriptional activation and tumor necrosis factor alpha production through RelA phosphorylation mediated by mitogen- and stress-activated protein kinase 1 in response to Borrelia burgdorferi antigens. Infect Immun.

[41] Saha RN (2007). MAPK p38 regulates transcriptional activity of NF-kappaB in primary human astrocytes via acetylation of p65. J Immunol.

[42] Pu Y (2020). Dual role of RACK1 in airway epithelial mesenchymal transition and apoptosis. J Cell Mol Med.

[43] Song S (2019). Foxm1 is a critical driver of TGF-β-induced EndMT in endothelial cells through Smad2/3 and binds to the Snail promoter. J Cell Physiol.

[44] Metwally H (2020). Noncanonical STAT1 phosphorylation expands its transcriptional activity into promoting LPS-induced IL-6 and IL-12p40 production. Sci Signal.

[45] Fearnley GW (2014). VEGF-A isoforms differentially regulate ATF-2-dependent VCAM-1 gene expression and endothelial-leukocyte interactions. Mol Biol Cell.

[46] Monnet E (2020). Efficacy and safety of NI-0101, an anti-toll-like receptor 4 monoclonal antibody, in patients with rheumatoid arthritis after inadequate response to methotrexate: a phase II study. Ann Rheum Dis.

[47] Isobe S (2019). Endothelial-mesenchymal transition drives expression of CD44 variant and xCT in pulmonary hypertension. Am J Respir Cell Mol Biol.

[48] Lemaitre C (2017). A human endogenous retrovirus-derived gene that can contribute to oncogenesis by activating the ERK pathway and inducing migration and invasion. PLoS Pathog.

[49] Hu X (2016). Expression of the env gene from the avian endogenous retrovirus ALVE and regulation by miR-155. Arch Virol.

[50] Gyamfi J (2018). Interleukin-6/STAT3 signalling regulates adipocyte induced epithelial-mesenchymal transition in breast cancer cells. Sci Rep.

[51] Hautefort A (2019). Bmpr2 mutant rats develop pulmonary and cardiac characteristics of pulmonary arterial hypertension. Circulation.

[52] Sun X (2020). Direct extracellular NAMPT involvement in pulmonary hypertension and vascular remodeling. Transcriptional regulation by SOX and HIF-2α. Am J Respir Cell Mol Biol.

[53] Mammoto T (2018). Twist1 in Hypoxia-induced pulmonary hypertension through transforming growth factor-β-Smad signaling. Am J Respir Cell Mol Biol.

[54] Singh K (2019). Cutaneous epithelial to mesenchymal transition activator ZEB1 regulates wound angiogenesis and closure in a glycemic status-dependent manner. Diabetes.

[55] Duperray A (2015). Inflammatory response of endothelial cells to a human endogenous retrovirus associated with multiple sclerosis is mediated by TLR4. Int Immunol.

[56] Bardin N (2001). Identification of CD146 as a component of the endothelial junction involved in the control of cell-cell cohesion. Blood.

[57] Mannam VK (2013). The fate of renal allografts hinges on responses of the microvascular endothelium. Exp Mol Pathol.

[58] Mlcochova P (2020). TLR4-mediated pathway triggers interferon-independent G0 arrest and antiviral SAMHD1 activity in macrophages. Cell Rep.

[59] Shatz-Azoulay H (2020). The animal lectin galectin-8 promotes cytokine expression and metastatic tumor growth in mice. Sci Rep.

[60] Andujar I (2010). Shikonin reduces oedema induced by phorbol ester by interfering with IkappaBalpha degradation thus inhibiting translocation of NF-kappaB to the nucleus. Br J Pharmacol.

[61] Wiley RD, Gummuluru S (2006). Immature dendritic cell-derived exosomes can mediate HIV-1 trans infection. Proc Natl Acad Sci U S A.

[62] Kadiu I (2012). Biochemical and biologic characterization of exosomes and microvesicles as facilitators of HIV-1 infection in macrophages. J Immunol.

[63] Roth WW (2015). Micro RNA in exosomes from HIV-infected macrophages. Int J Environ Res Public Health.

[64] Mukhamedova N (2019). Exosomes containing HIV protein Nef reorganize lipid rafts potentiating inflammatory response in bystander cells. PLoS Pathog.

[65] Chiappinelli KB (2015). Inhibiting DNA methylation causes an interferon response in cancer via dsRNA including endogenous retroviruses. Cell.

[66] Canadas I (2018). Tumor innate immunity primed by specific interferon-stimulated endogenous retroviruses. Nat Med.

[67] Deng L (2016). Characterization and functional studies of fowl adenovirus 9 dUTPase. Virology.

[68] Omura J (2016). Protective roles of endothelial AMP-activated protein kinase against hypoxia-induced pulmonary hypertension in mice. Circ Res.

[69] Savale L (2014). Pulmonary arterial hypertension in patients treated with interferon. Eur Respir J.

[70] Otsuki S (2015). Potential contribution of phenotypically modulated smooth muscle cells and related inflammation in the development of experimental obstructive pulmonary vasculopathy in rats. PLoS One.

[71] Elias JE, Gygi SP (2007). Target-decoy search strategy for increased confidence in large-scale protein identifications by mass spectrometry. Nat Methods.

[72] Miyagawa K (2019). Smooth muscle contact drives endothelial regeneration by BMPR2-Notch1-mediated metabolic and epigenetic changes. Circ Res.

