# Supplementary material for: Monocyte-released HERV-K dUTPase engages TLR4 and MCAM causing endothelial mesenchymal transition
Source: JCI Insight. 2021 Aug 9;6(15):e146416. doi: 10.1172/jci.insight.146416 (PMC8410063; doi:10.1172/jci.insight.146416)
Supplement: Supplemental data [file jciinsight-6-146416-s103.pdf]

Supplemental Material

Monocyte Released HERV-K dUTPase Engages TLR4 and MCAM Causing Endothelial Mesenchymal Transition

Shoichiro Otsuki[1]; Toshie Saito[1]; Shalina Taylor[1]; Dan Li[1]; Jan-Renier Moonen[1]; David P Marciano[1]; Rebecca L Harper[1]; Aiqin Cao[1]; Lingli Wang[1]; Maria E Ariza[2]; Marlene Rabinovitch[1*]

[1]Department of Pediatrics, Division of Cardiology, and Cardiovascular Institute, Stanford University School of Medicine, Stanford, CA, United States

[2]Department of Cancer Biology and Genetics and Institute for Behavioral Medicine Research, The Ohio State University Wexner Medical Center, Columbus, OH, United States

Supplemental Data

Table S1. Demographics and other characteristics of donors

ID	Age (y)	Sex	Race	Ethnicity	Cause of Death
**Donor 1**	14	M	White	Non-Hispanic	Gun Shot Wound to head
**Donor 2**	41	F	White	Non-Hispanic	Cerebrovascular accident
**Donor 3**	56	F	White	Non-Hispanic	Cerebrovascular accident

Table S2. Demographics and other characteristics of PAH patients

ID	Age (y)	Sex	Race	Ethnicity	Diagnosis	BMPR2 Mut.[1]	PAP[2] s/d/m	PVR[3] (WU)	6MW[4] (m)	PAH medication
**PAH1**	41	F	White	Non-Hispanic	IPAH	No	75/43/55	9.81	472	Sildenafil<br>Bosentan<br>IV epoprostenol
**PAH2**	51	M	White	Non-Hispanic	IPAH	No	41/19/30	6.09	378	Sildenafil<br>IV epoprostenol

[1]BMPR2 Mut., BMPR2 mutation

[2]PAP s/d/m, Pulmonary Artery Pressure (systolic/diastolic/mean); value closest to transplant

[3]PVR, Pulmonary Vascular Resistance

[4]6MW, distance walked during six minutes; value closest to transplant

Table S3. Primers used for qPCR

Gene	Forward primer	Reverse Primer
SNAIL	TACAGCGAGCTGCAGGACT	ATCTCCGGAGGTGGGATG
SLUG	TGGTTGCTTCAAGGACACAT	GTTGCAGTGAGGGCAAGAA
CDH5	GTTCACCTTCTGCGAGGATA	GTAGCTGGTGGTGTCCATCT
PECAM1	GCAACACAGTCCAGATAGTCGT	GACCTCAAACTGGGCATCAT
ACTA2	CCCTGAAGTACCCGATAGAACA	GGCAACACGAAGCTCATTG
SM22	TTCCAGACTGTTGACCTCTTTG	CAAAGCCATCAGGGTCCTC
HERV-K dUTPase	AAATGGGCAACCATTGTCGGGAAACGAGC	TAGTACATAAATCTACTGCTGCACTGC
TLR4	AGACCTGTCCCTGAACCCTAT	CGATGGACTTCTAAACCAGCCA
TLR3	TTGCCTTGTATCTACTTTTGGGG	TCAACACTGTTATGTTTGTGGGT
TLR2	ATCCTCCAATCAGGCTTCTCT	GGACAGGTCAAGGCTTTTTACA
TLR6	AGACCTACCGCTGAAAACCAA	ACTCACAATAGGATGGCAGGA
BMPR2	CGGCTGCTTCGCAGAATCA	TCTTGGGGATCTCCAATGTGAG
VCAM1	GGGAAGATGGTCGTGATCCTT	TCTGGGGTGGTCTCGATTTTA
IL6	ACTCCCTCTTCAGAACGAATTG	CCATCTTTGCAAGGTTCAGGTTG
MYD88	GGCTGCTCTCAACATGCGA	CTGTGTCCGCACGTTCAAGA
CD36	GGCTGTGACCGGAACTGTG	AGGTCTCCAACTGGCATTAGAA
NRP1	GGCGCTTTTCGCAACGATAAA	TCGCATTTTTCACTTGGGTGAT
ENG	TGCACTTGGCCTACAATTCCA	AGCTGCCCACTCAAGGATCT
MCAM	AGCTCCGCGTCTACAAAGC	CTACACAGGTAGCGACCTCC
p65	ATGTGGAGATCATTGAGCAGC	CCTGGTCCTGTGTAGCCATT
SMAD3	TGGACGCAGGTTCTCCAAAC	CCGGCTCGCAGTAGGTAAC
STAT1	CAGCTTGACTCAAAATTCCTGGA	TGAAGATTACGCTTGCTTTTCCT
ATF2	AATTGAGGAGCCTTCTGTTGTAG	CATCACTGGTAGTAGACTCTGGG
β-Actin	CATGTACGTTGCTATCCAGGC	CTCCTTAATGTCACGCACGAT

Table S4. Antibodies used for Western immunoblotting

Antibody	Dilution	Company
β-Actin	1:1000	Santa Cruz, Cat#47778
GAPDH	1:1000	Santa Cruz, Cat#365062
Lamin-B1	1:500	Santa Cruz, Cat#30264
Histone 3	1:500	Santa Cruz, Cat#517576
SNAIL/SLUG	1:500	Abcam, Cat#180714
CDH5	1:1000	Abcam, Cat#33168
PECAM1	1:1000	Abcam, Cat#9498
ACTA2	1:500	Sigma-Aldrich, Cat#A2547
VCAM1	1:1000	Abcam, Cat#134047
TLR4	1:1000	Santa Cruz, Cat#293072
MCAM	1:1000	Cell Signaling, Cat#13475
p-IκBα	1:1000	Cell Signaling, Cat#2859
IκBα	1:1000	Cell Signaling, Cat#4814
p-p65	1:1000	Abcam, Cat#106129
p65	1:1000	Cell Signaling, Cat#8242
p-p38	1:1000	Cell Signaling, Cat#9215
p-JNK	1:1000	Santa Cruz, Cat#6254
p-ERK1/2	1:1000	Cell Signaling, Cat#9101
p-SMAD2	1:1000	Cell Signaling, Cat#3108
p-SMAD3	1:1000	Cell Signaling, Cat#9520
SMAD2/3	1:1000	Cell Signaling, Cat#3102
p-STAT1	1:1000	Cell Signaling, Cat#9167
STAT1	1:1000	Cell Signaling, Cat#9172
p-ATF2	1:1000	Santa Cruz, Cat#8398
ATF2	1:200	Santa Cruz, Cat#242
CD9	1:1000	Invitrogen, Cat#10626D
HERV-K dUTPase	10 µg/ml	Dr. Ariza, Ohio State University

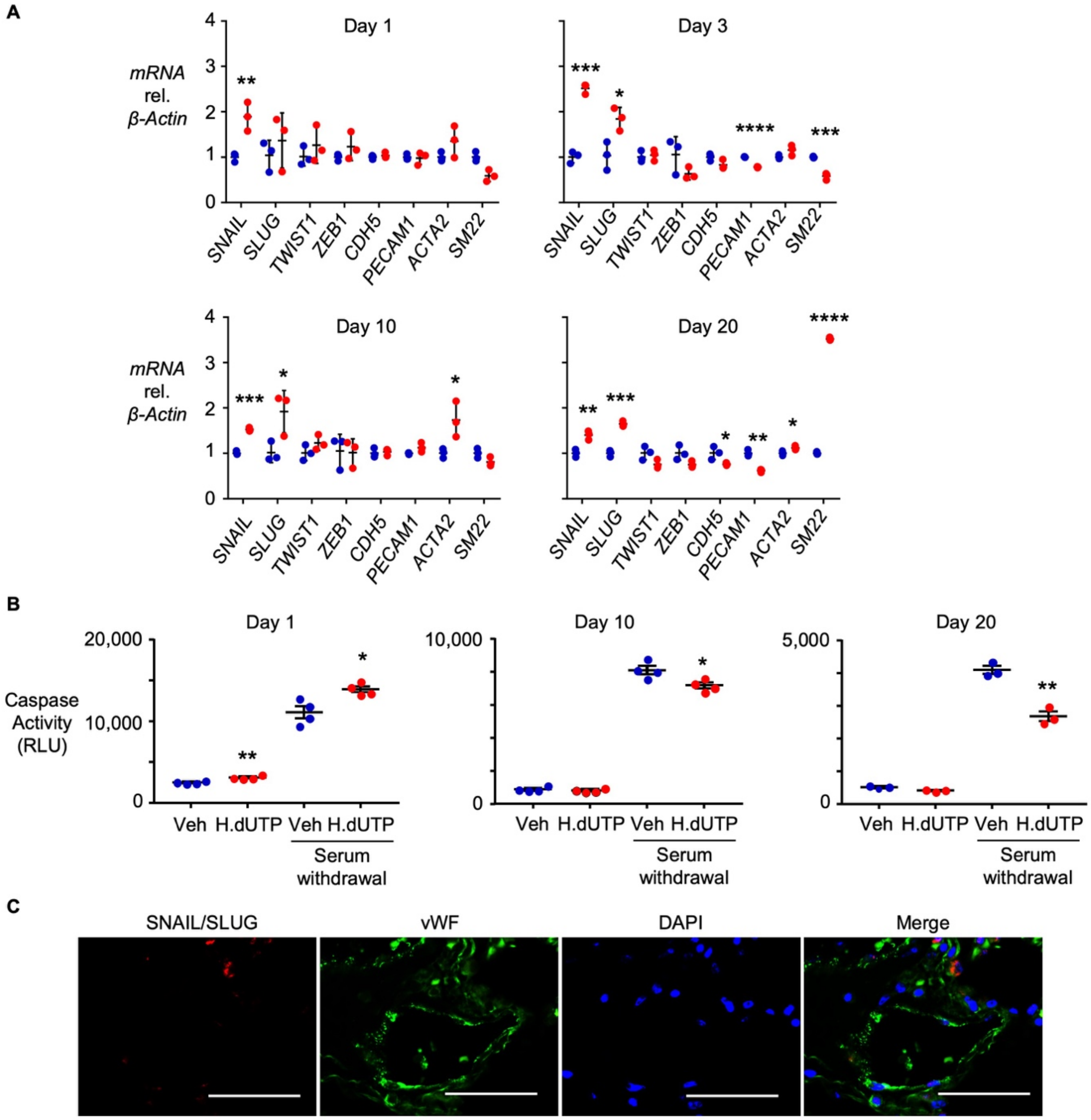

Figure S1. Time Course for induction of SNAIL and features of EndMT and apoptosis in PAECs by recombinant HERV-K dUTPase and lack of SNAIL-SLUG detection in control human pulmonary arteries.

(A) Commercially available PAECs were treated with 10 µg/mL of HERV-K dUTPase or PBS vehicle daily for 3 days, then every 3 days for up to 20 days. PAECs were used at passage 3 to 8. Gene expression changes (HERV-K dUTPase vs. Vehicle) in EndMT, EC, and SMC markers were assessed by qPCR at 1, 3, 10, and 20 days, and normalized to β-Actin. Individual data points are represented as blue circles for PAECs treated with vehicle or red circles for PAECs treated with HERV-K dUTPase, and shown with n=3 mean ± SEM. $^*p<0.05$; $^{**}p<0.01$; $^{***}p<0.001$; $^{****}p<0.0001$ vs. Veh by unpaired Student t test. **(B)** Propensity to apoptosis is judged in response to control vehicle or HERV-K dUTPase after 16h of serum withdrawal on day 1, day 10 and day 20. Individual data points are represented as in (A). n=4, mean±SEM $^*p<0.05$ and $^{**}p<0.01$ vs. veh under the same condition, by unpaired Student t-test. **(C)** Representative confocal images of a PA of a healthy Control stained for the EndMT transcription marker SNAIL/SLUG(red), the endothelial marker vWF (green), and DAPI (blue). Scale bar, 20 µm.

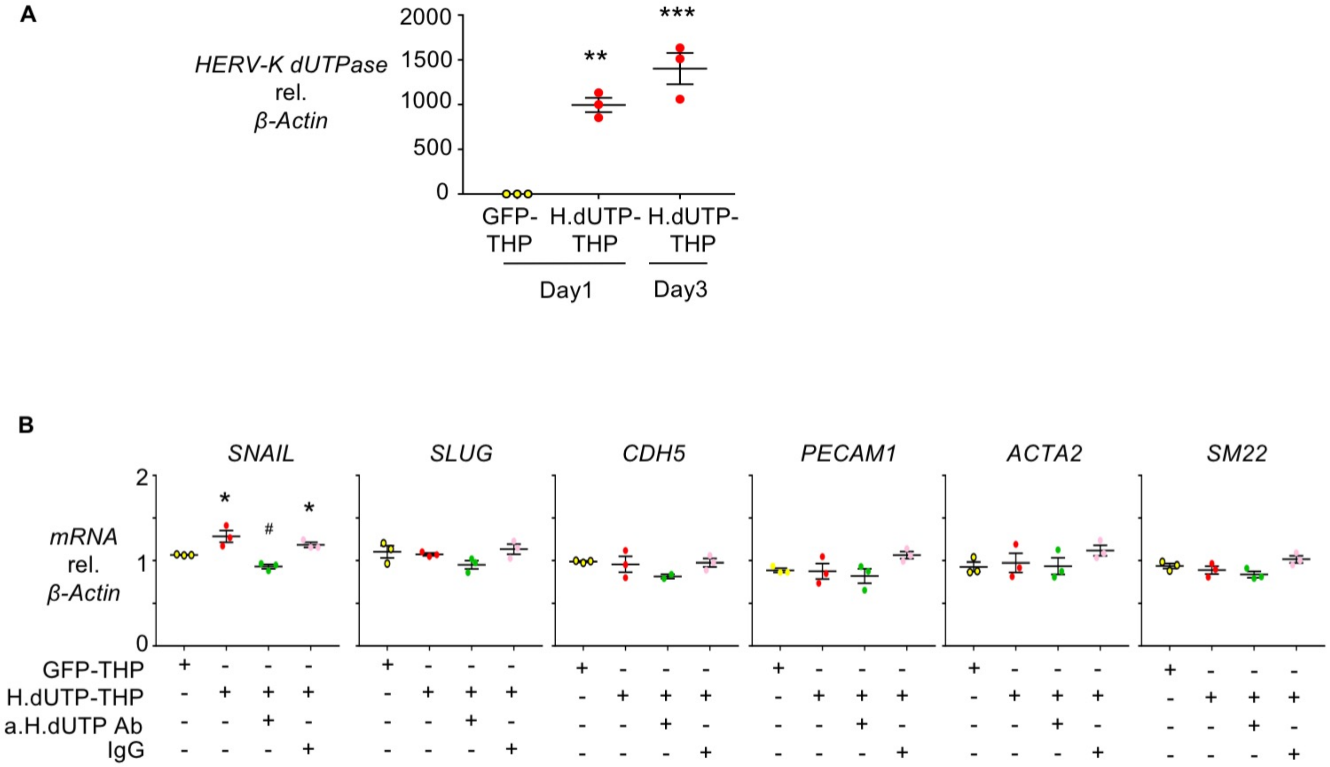

Figure S2. Sustained expression of HERV-K dUTPase in THP-1 cells and in Co-culture with PAECs, SNAIL is induced as early as Day 1.

(**A**) PAECs were co-cultured with THP-1 monocytes transfected with HERV-K dUTPase or GFP (H.dUTP-THP or GFP-THP, respectively) using a trans-well system with a 1 μm pore membrane, as described in Figure 6. Gene expression levels of *HERV-K dUTPase* in GFP-THP and H.dUTP-THP monocytes were assessed after 1 and 3 days in co-culture. (**B**) Gene expression levels of EndMT, EC, and SMC markers in PAECs treated for 1 day were assessed by qPCR. Data are expressed as fold-change compared with GFP-THP, and shown n=3, mean ± SEM. *p<0.05; **p<0.01; ***p<0.001 vs. GFP-THP, #p<0.05; ##p<0.01 vs. H.dUTP-THP, by a one-way ANOVA followed by Tukey multiple comparison test.

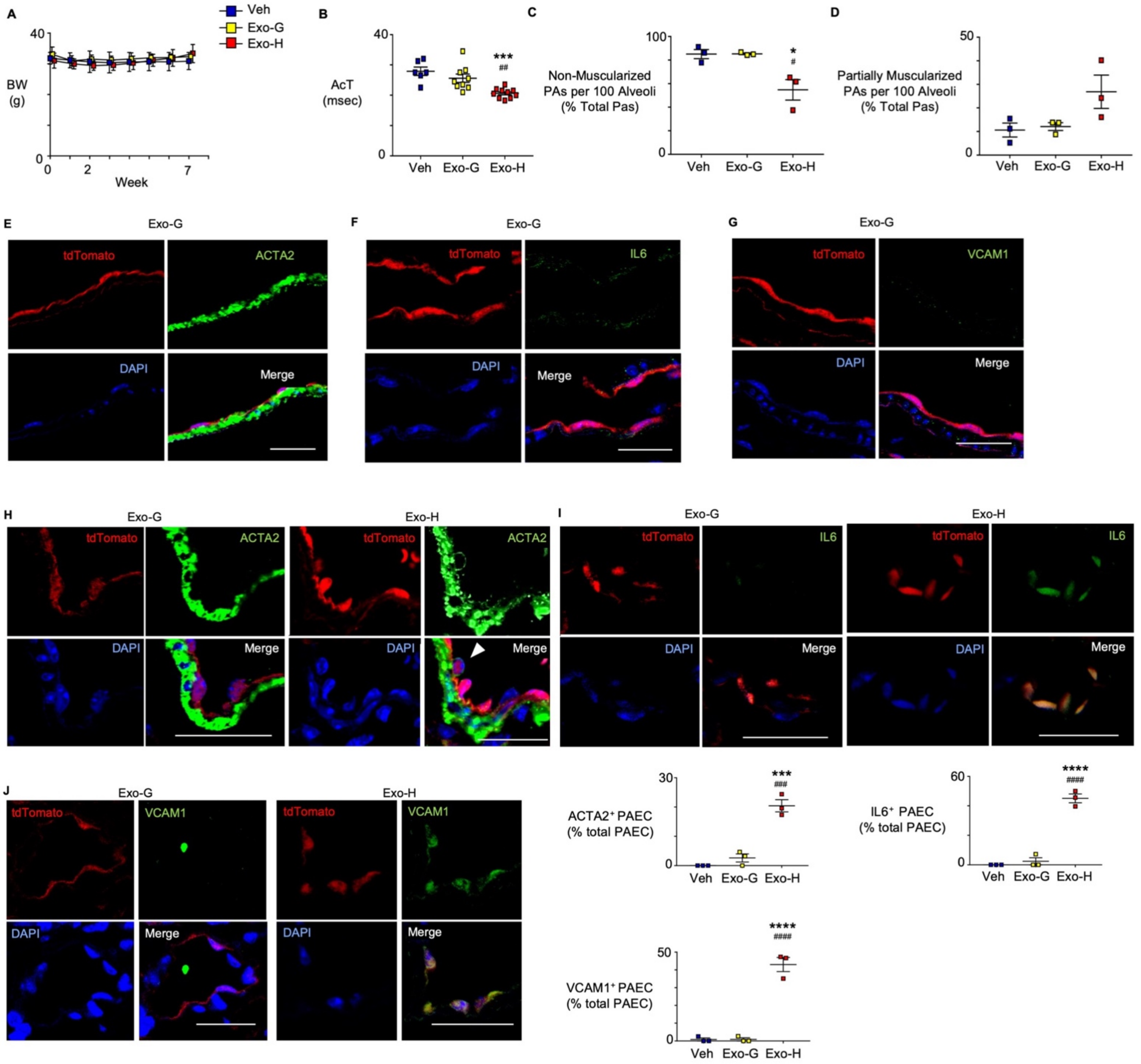

Figure S3. EVs from THP-1 monocytes containing HERV-K dUTPase induce pulmonary hypertension, judged by Acceleration Time (AcT), and a reduction in non-muscular and an increase in partially distal pulmonary arteries.

(**A**) Body weight of mice receiving extracellular vesicles (EVs) from medium of THP-1 monocytes overexpressing HERV-K dUTPase (Exo-H, n=9) or GFP (Exo-G, n=9), and as control, from mice administered PBS vehicle (Veh, n=6). (**B-D**) Mice treated with Exo-H (n=9) vs. Exo-G) (n=9), or vehicle control (n=6) exhibit **(B)** decreased PA acceleration time (AcT), **(C)** decreased proportion of non-muscularized and **(D)** a trend toward increased proportion of partially muscularized distal PAs compared with control mice. (n=3 per group randomly selected). **(E, F and G)** Exo-G large artery controls are shown as quantified in **main text Figures 3 F, G and H**. **(H)** Exo-G vs. Exo-H small PAs showing ACTA 2 (EndMT),**)** **(I)** IL6**,** and **(J)** VCAM1. n=3, mean±SEM. *$p<0.05$; ***$p<0.001$ vs. Veh, #$p<0.05$; ##$p<0.01$ vs. Exo-G, by one-way ANOVA followed by Tukey multiple comparison test.

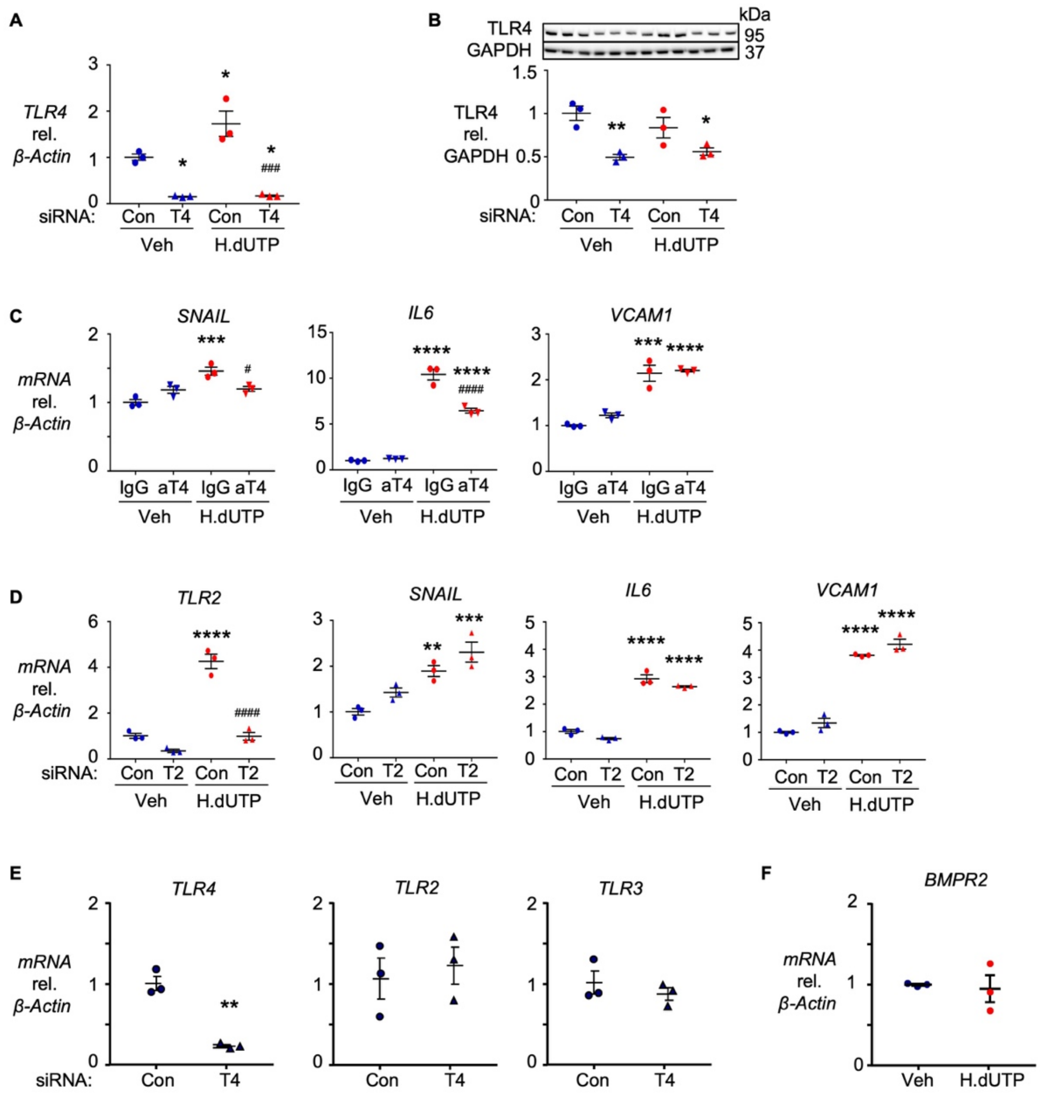

Figure S4. TLR4, but not TLR2, mediates HERV-K dUTPase response.
(**A, B**) PAECs were transfected with siRNA targeting TLR4 (T4) or with nontargeting siRNA (Con) for 48 hours, before treatment with HERV-K dUTPase (d.UTP) or vehicle (Veh) for 72 hours as described in Figure 2. TLR4 gene and protein expression were assessed by qPCR, immunoblot and densitometric quantification. (**C**) PAECs were pretreated with 10 µg/mL of neutralizing antibody for TLR4 (aT4) or with isotype IgG (IgG) for one hour, followed by treatment every day for 3 days with 10 µg/mL of HERV-K dUTPase or vehicle. *SNAIL*, *IL6*, and *VCAM1* gene expression changes were assessed by qPCR. (**D**) PAECs were transfected with siRNA targeting TLR2 (T2) or with nontargeting siRNA (Con) for 48 hours before 10 µg/mL HERV-K dUTPase treatment for 72 hours, and gene expression changes were assessed by qPCR. Data are expressed as fold-change compared with Veh/Con (**A**, **B**, and **D**) or Veh/IgG (**C**), and show n=3, mean ± SEM. *p<0.05; **p<0.01; ***p<0.001; ****p<0.0001 vs. Veh/Con or Veh/IgG, and #p<0.05; ###p<0.001; ####p<0.0001 vs. H.dUTP/Con or H.dUTP/IgG by a one-way ANOVA followed by Tukey multiple comparison test. **(E)** Reduction of *TLR4* and resulting *TLR2* and *TLR3 mRNA* expression. **(F)** HERV-K dUTPase and *BMPR2* levels as assessed on day 3. In (**E, F**), n=3, mean ± SEM, fold-change compared with Con, analyzed by Student t-test.

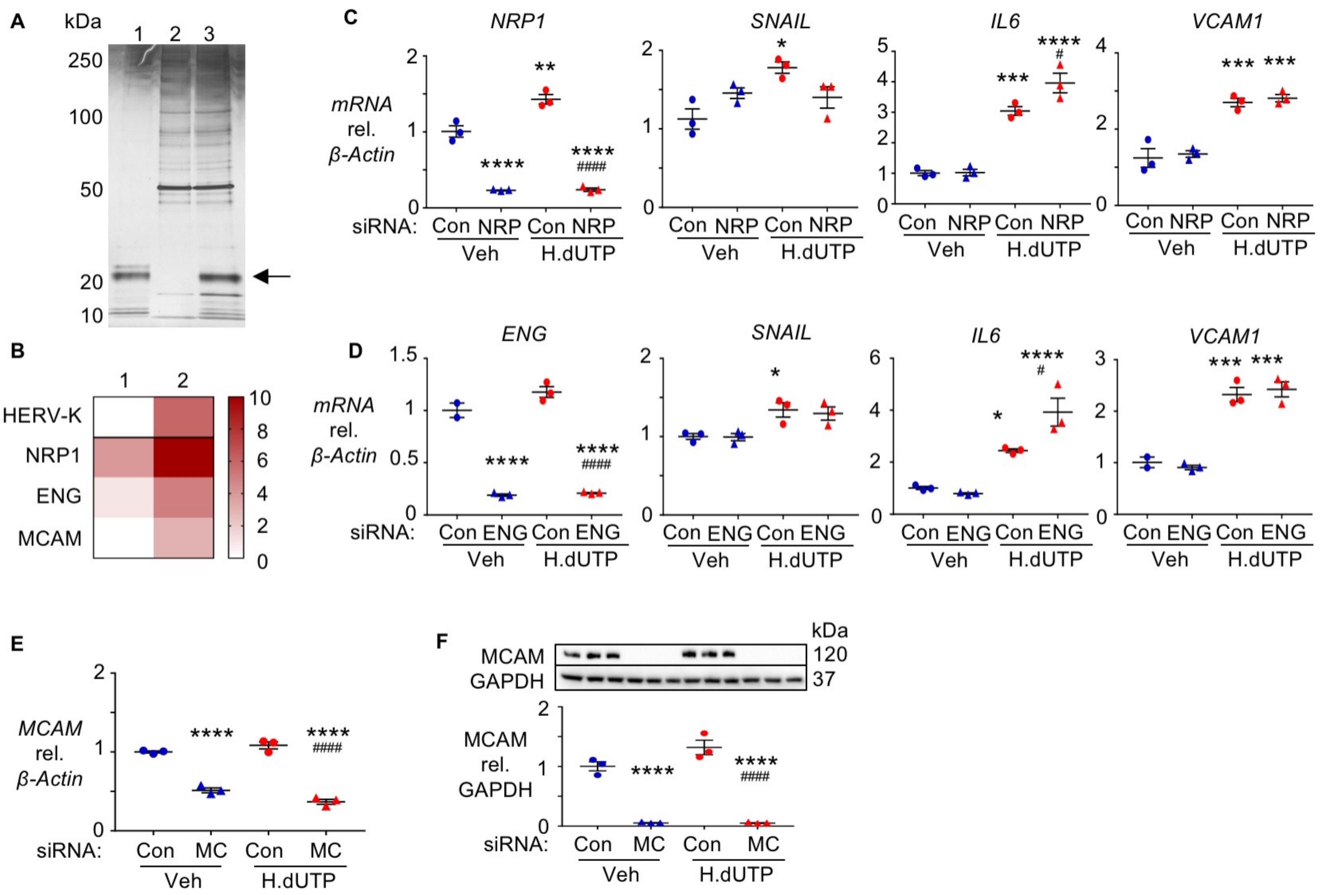

Figure S5. NRP1 and ENG associate with HERV-K dUTPase but do not mediate HERV-K dUTPase EndMT and MCAM siRNA results in an efficient reduction in mRNA and protein.

(**A**) Candidate receptors for HERV-K dUTPase from affinity purification followed by mass spectrometry. The poly-histidine pull-down technique was used to identify putative binding partners of HERV-K dUTPase, with a membrane protein extracted from PAECs was used as 'prey'. Lane 1, silver stain of purified HERV-K dUTPase control. Arrow denotes band. Lane 2, non-treated gel control (minus histidine-tagged HERV-K dUTPase) plus prey proteins; and lane 3, purified HERV-K dUTPase-protein interaction (histidine-tagged HERV-K dUTPase and prey proteins). (**B**) Heatmap of mass spectrometry showing interacting receptors and other proteins. Non-treated gel control (lane 1) vs. purified HERV-K dUTPase-protein interaction (lane 2). (**C and D**) PAECs were transfected with siRNA targeting neuropilin-1 (*NRP1*) or endoglin (*ENG*), or with nontargeting siRNA (Con) 48 hours before 10 µg/mL HERV-K dUTPase treatment for 72 hours, and *SNAIL*, *IL6*, and *VCAM1* gene expression changes were assessed by qPCR. (**E and F**) PAECs were transfected with siRNA for MCAM (MC) or nontargeting siRNA (Con) 48 hours before addition of 10 µg/mL HERV-K dUTPase, and MCAM gene and protein expression assessed by qPCR, immunoblot and densitometric quantification 72 hours after daily 10 µg/mL of HERV-K dUTPase or vehicle treatment. In C-F, data are expressed as fold-change compared to Veh/Con, and show n=3, mean ± SEM. *p<0.05; **p<0.01; ***p<0.001; ****p<0.0001 vs. Veh/Con, and #p<0.05; ####p<0.0001 vs. H.dUTP/Con by a one-way ANOVA followed by Tukey multiple comparison test.

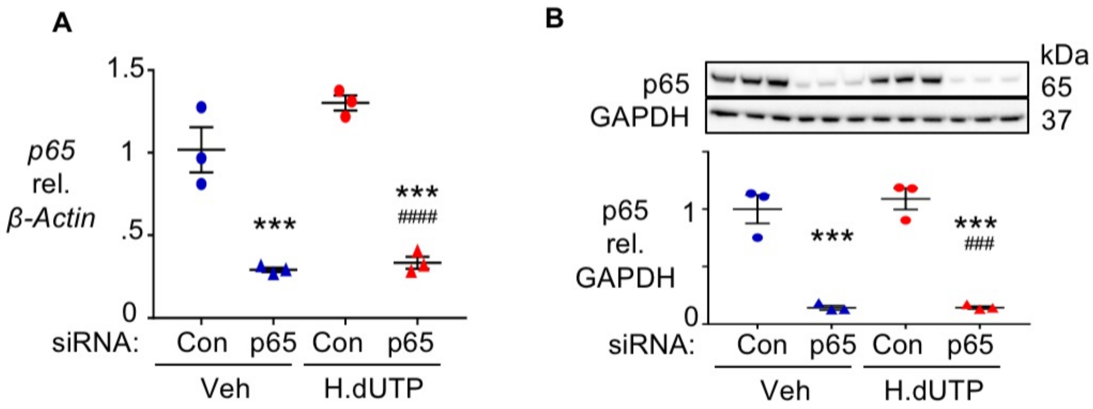

Figure S6. siRNA targeting p65 effectively suppresses the expression of p65.

PAECs were transfected with siRNA targeting p65 or with nontargeting siRNA (Con) for 48 hours before addition of 10 µg/mL HERV-K dUTPase. (**A**) p65 gene expression assessed by qPCR, and (**B**) protein expression assessed by immunoblot and densitometric quantification 72 hours after daily 10 µg/mL of HERV-K dUTPase or vehicle treatment. Data are expressed as fold-change compared with Veh/Con, and show n=3, mean ± SEM. ***$p<0.001$ vs. Veh/Con, and ###$p<0.001$; ####$p<0.0001$ vs. H.dUTP/Con by a one-way ANOVA followed by Tukey multiple comparison test.

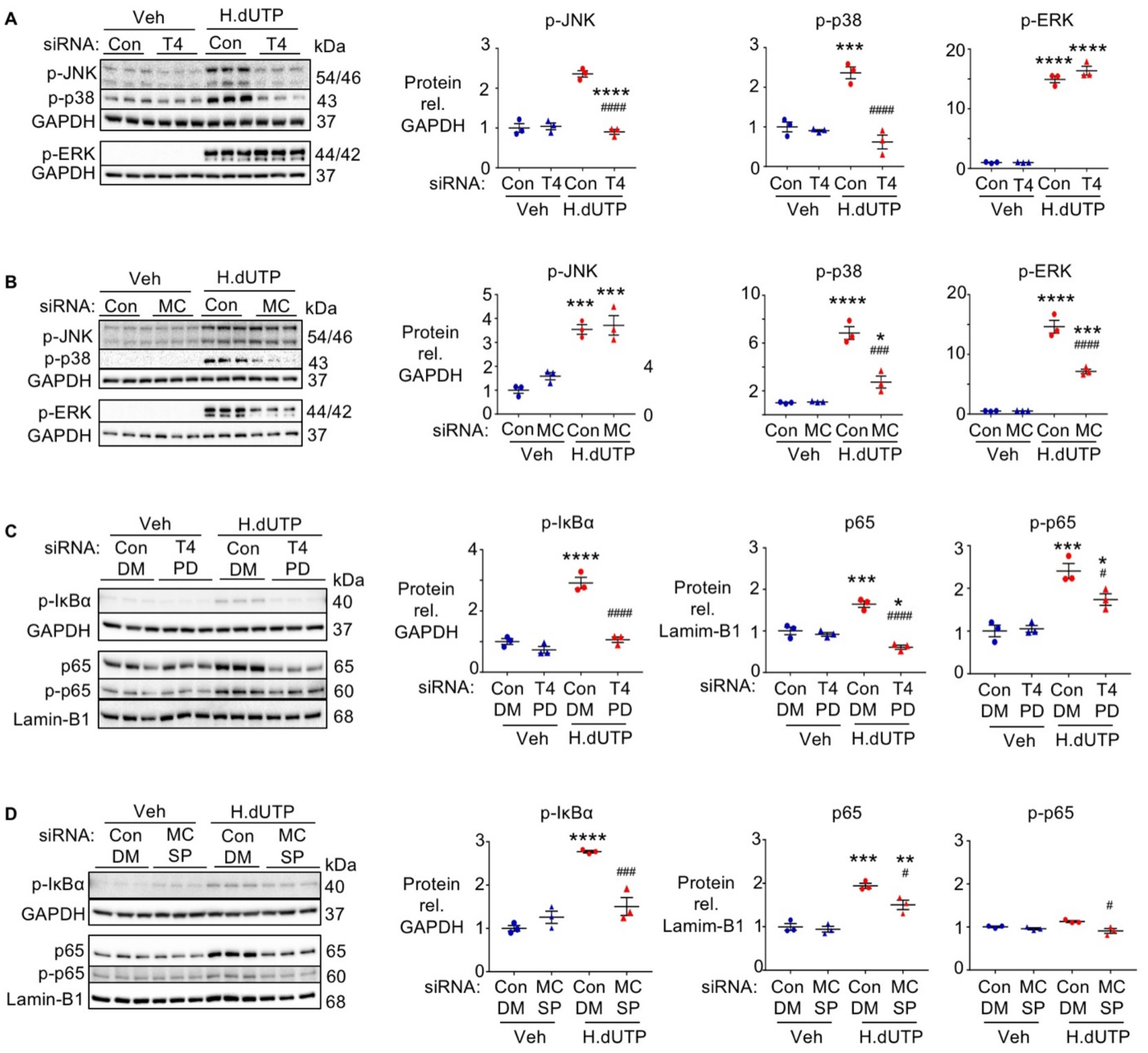

Figure S7. TLR4 and MCAM mediate the phosphorylation of p38, and p65 nuclear translocation rescued by pERK and pJNK with loss of TLR4 or MCAM.

(**A and B**) PAECs were transfected with siRNA targeting TLR4 (T4) (**A**) or MCAM (MC) (**B**) or with nontargeting siRNA (Con) for 48 hours before HERV-K dUTPase treatment for 4 hours, and p-p38, p-JNK, and p-ERK protein expression were assessed by immunoblot and densitometric quantification. (**C**) PAECs were transfected with siRNA targeting TLR4 (T4) or with nontargeting siRNA (Con), followed by treatment with the ERK inhibitor PD98059 (PD; 20 μM) or DMSO (DM) for 2 hours, prior to HERV-K dUTPase treatment for 1 hour. Cytoplasmic p-IκBα, and nuclear p-p65 and p65 protein expression were assessed by immunoblot and densitometric quantification. (**D**) PAECs were transfected with siRNA targeting MCAM (MC) or with nontargeting siRNA (Con), followed by treatment with the JNK inhibitor SP600125 (SP; 30 μM) or DMSO (DM) for 45 minutes, prior to HERV-K dUTPase treatment for one hour. Cytoplasmic p-IκBα, and nuclear p-p65 and p65 protein expression were assessed by immunoblot and densitometric quantification. Data are expressed as fold-change compared with Veh/Con (A and B) or Veh/Con/DM (C and D), and show n=3, mean ± SEM. *p<0.05; **p<0.01; ***p<0.001; ****p<0.0001 vs. Veh/Con or Veh/Con/DM, and #p<0.05; ##p<0.01; ###p<0.001; ####p<0.0001 vs. H.dUTP/Con or H.dUTP/Con/DM by a one-way ANOVA followed by Tukey multiple comparison test.

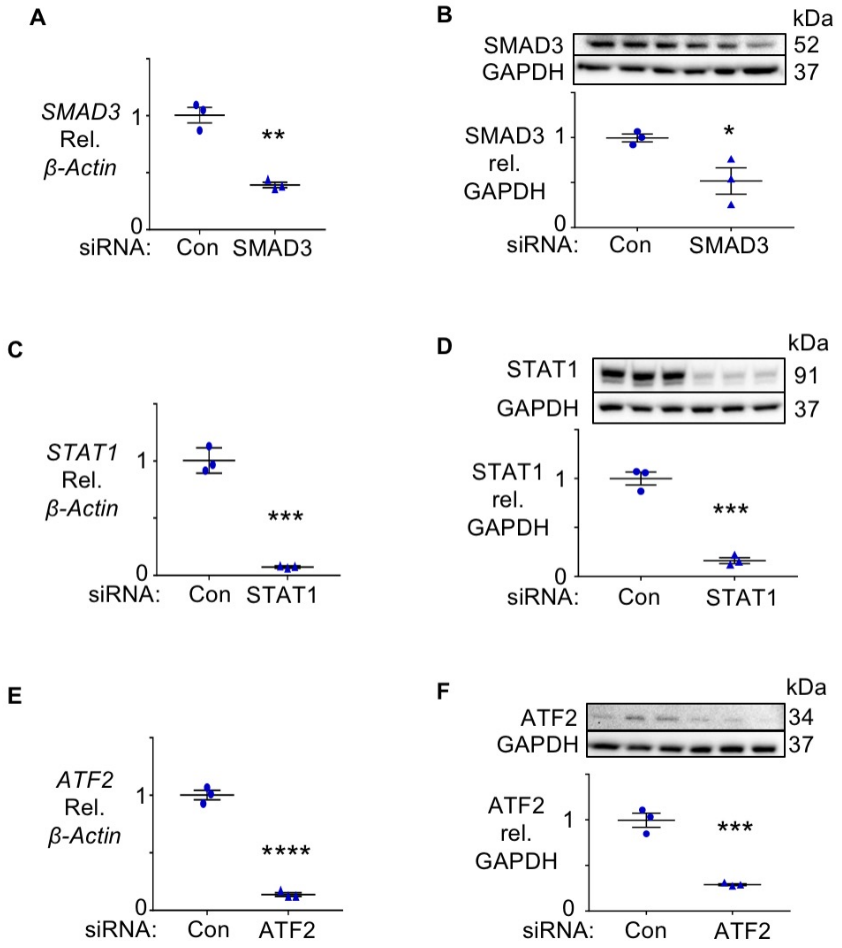

Figure S8. siRNA targeting SMAD3, STAT1 or ATF2 suppressed the target gene and protein expression.

PAECs were transfected with siRNA for SMAD3 (**A and B**), STAT1 (**C and D**) or ATF2 (**E and F**) or with nontargeting siRNA (Con) for 48 hours, and target gene and protein expression were assessed by qPCR, immunoblot and densitometric quantification. Data are expressed as fold-change compared with Con, and show n=3, mean ± SEM. *$p<0.05$; **$p<0.01$; ***$p<0.001$; ****$p<0.0001$ vs. Con by unpaired Student t test.
